# Inhibition of Accumulation of Neutral Lipids and Their Hydroperoxide Species in Hepatocytes by Bioactive *Allium sativum* Extract

**DOI:** 10.3390/antiox13111310

**Published:** 2024-10-28

**Authors:** Dya Fita Dibwe, Saki Oba, Satomi Monde, Shu-Ping Hui

**Affiliations:** 1 Faculty of Health Sciences, Hokkaido University, Kita-12, Nishi-5, Kita-Ku, Sapporo 060-0812, Japan; dibwedf@hs.hokudai.ac.jp; 2 Graduate School of Health Sciences, Hokkaido University, Kita-12, Nishi-5, Kita-Ku, Sapporo 060-0812, Japan; saki.oba.h@gmail.com (S.O.); monde.satomi.p3@elms.hokudai.ac.jp (S.M.)

**Keywords:** functional foods, nutraceutical, hepatosteatosis, oxidative lipidomics, triacylglycerols

## Abstract

Our ongoing research suggests that extracts from plant-based foods inhibit the accumulation of lipid droplets (LDs) and oxidized lipid droplets (oxLDs) in liver cells. These findings suggest their potential use in the alleviation of metabolic dysfunction-associated fatty liver disease (MAFLD) and its most severe manifestation, metabolic dysfunction-associated steatohepatitis (MASH). *Allium* extracts (ALs: AL1–AL9) were used to assess their ability to reduce lipid droplet accumulation (LDA) and oxidized lipid droplet accumulation (oxLDA) by inhibiting neutral lipid accumulation and oxidation in LD. Among the tested *Allium* extracts, AL1, AL3, and AL6 demonstrated substantial inhibitory effects on the LDA. Furthermore, AL1 extract showed real-time inhibition of LDA in HepG2 cells in DMEM supplemented with oleic acid (OA) within 12 h of treatment. Our lipidomic approach was used to quantify the accumulation and inhibition of intracellular triacylglycerol (TAG) and oxidized TAG hydroperoxide [TG (OOH) n = 3] species in hepatocytes under OA and linoleic acid loading conditions. These results suggest that *Allium*-based foods inhibit LD accumulation by decreasing intracellular lipids and lipid hydroperoxides in the hepatocytes. The metabolomic analysis of AL1—the bioactive LDAI extract—using both LC-MS/MS and 1D-NMR [^1^H, ^13^C, and Dept (135 and 90)] approaches revealed that AL1 contains mainly carbohydrates and glucoside metabolites, including iridoid glucosides, as well as minor amino acids, organosulfur compounds, and organic acids such as the antioxidant ascorbic acid (KA2 = S**13**), and their derivatives, suggesting that AL1 could be a potential resource for the development of functional foods and in drug discovery targeting MAFLD/MASH and other related diseases.

## 1. Introduction

Chronic diseases frequently arise from inflammation-induced oxidative stress, which leads to the accumulation of reactive oxygen species (ROS) that can harm cellular components, including DNA, proteins, and lipid membranes. Excessive production of free radicals can damage essential macromolecules, including nucleic acids, proteins and lipids, leading to tissue deterioration associated with many chronic and degenerative diseases. Free radicals cause oxidative damage to biomolecules. The hydroxyl radical is exceptionally reactive and can quickly remove hydrogen atoms from biomolecules in its immediate vicinity [1]. The primary targets of hydroxyl radicals in lipids are phospholipids and membrane triglycerides [2,3]. The oxidation of lipid radicals results in lipid peroxidation, which is linked to the accumulation of neutral lipids and their oxidized species within cells. The accumulation of these lipids has been associated with several human metabolic diseases such as MAFLD/MASH [4,5]. MAFLD is defined as excessive accumulation of free fatty acids (FFAs) in the liver. The accumulation of FFAs by hepatocytes in the bloodstream is significant, leading to the presence of both local and circulating FFAs. As reported in several studies, FFAs may cause various negative effects in humans, such as insulin resistance, hyperlipidemia, inflammation, and hepatic lipidosis [6,7,8,9]. Lipotoxicity plays a significant role in the emergence of MAFLD. Numerous studies have found a correlation between surplus intracellular LD accumulation and obesity, diabetes, and other metabolic disorders [10,11]. Hepatic LDA is believed to play a role in the preliminary stages of MAFLD [12,13]. The primary fatty acids found in the human body are palmitic and oleic acids, which can easily be transformed into neutral lipid triacylglycerol (TAG) and its oxidized form, triacylglycerol hydroperoxide (TGOOH). Understanding the roles of these substances in the uptake of intracellular components and cytotoxicity is essential to understand the mechanisms of MASH [12]. Therefore, a strategy for preventing and managing NAFLD/NASH is urgently required. Agents that can reduce LDA in hepatocytes and the liver are promising candidates for preventing and managing obesity-associated NAFLD [12,13,14]. A large number of studies have demonstrated a connection between the accumulation of intracellular lipid droplets (LDs) and various metabolic conditions, including obesity and diabetes [10,11]. Studies have also suggested that hepatic LDA may be involved in the early stages of MAFLD [15]. 

**Scheme 1:**
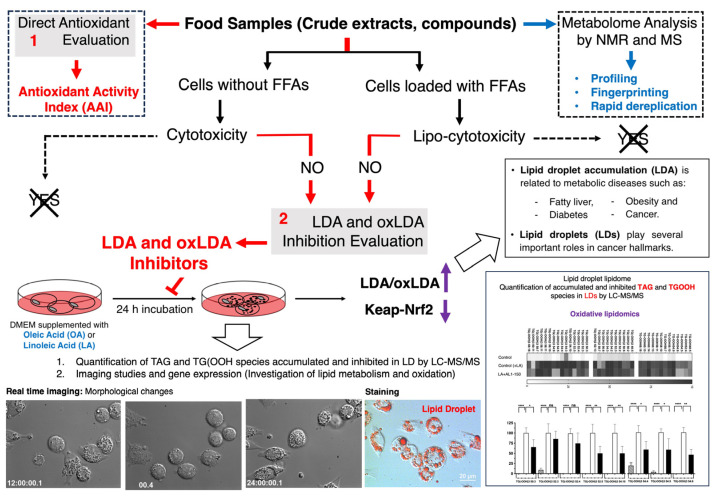
Discovery strategy of antioxidants and lipid droplet accumulation inhibitors from food origin. (1) Direct antioxidant and antioxidant activity index. (2) LDAI/oxLDAI under fatty acid-loaded conditions associated with Keap1-Nrf2 regulation.

Functional foods and nutraceuticals, which include natural products that can help decrease the accumulation of lipid droplets (LDs) and oxidized lipid droplets (oxLDs) in liver cells by controlling neutral lipids TAG and TGOOH, are hypothesized to be abundant in nature and show great potential for the prevention and drug discovery of MAFLD/MASH and related diseases, as demonstrated in previous studies [16,17,18,19,20,21,22,23,24,25,26]. Food-derived natural bioactive products with LDAI activity in hepatocytes are promising therapeutic candidates. In our ongoing studies, we have developed and explored a strategy for identifying antioxidants and LDAI/oxLDAI candidates from natural product extracts and food-derived compounds (Scheme 1). Utilizing natural resources rich in bioactive substances that exhibit antioxidant and LDA-inhibitory activities in liver cells presents a promising avenue for potential preventive and therapeutic applications. These bioactive extracts and compounds have emerged as promising candidates for MAFLD/MASH management. Functional foods contain potential secondary metabolites that provide health benefits in addition to basic nutrition. These products not only deliver essential nutrients and energy but also positively affect specific physiological functions, enhance beneficial responses, and lower disease risk [18]. Conversely, nutraceuticals are combinations of substances extracted from food or food components that offer medical or health advantages, including disease prevention and treatment [19].

The nature of the hydroperoxides formed depends on the oxidation method used. These initial reactions trigger a continuous chain reaction [12,27,28]. Our ongoing studies aim to address the limitations of conventional approaches to the analysis of oxidized lipids in LD lipidomes by focusing on advanced oxidative lipidomics techniques and strategies. Typically, assessment of oxidized lipids involves indirect detection of lipid oxidation products, which can be quantified by colorimetric assays, immunoassays or electron spin resonance spectroscopy. Common methods include assays for thiobarbituric acid reactive substances (TBARSs) and malondialdehyde (MDA), ferrous oxidation in xylenol orange (FOX), and conjugated diene tests. Although these techniques, including recent fluorescence-based methods, are commonly used to assess the scale of lipid oxidation, they often fail to provide comprehensive analyses of changes in specific lipid species [14,27,28,29,30]. To address this gap, the identification of oxidized molecular lipid species using MS/MS-based approaches has demonstrated considerable potential [14,27,28,29,30].

The prevention of hepatic steatosis relies primarily on the inhibition of LDA and lipid peroxidation. Our ongoing research has identified plant food extracts and secondary metabolites with LDAI activity, which led to the discovery of flazin, an alkaloid beta-carboline with a C-3 carboxyl group and a C-1 furfuryl alcohol moiety, and its derivative with a piperidine C-ring, from oyster extracts. These molecules show significant LDAI activity by regulating LD accumulation. Plant food extracts and bioactive LDAI compounds inhibit TAG species accumulation in cells, activate lipolysis, and suppress lipogenesis. These results suggest that plant food extracts and β-carboline alkaloids may be useful in preventing MAFLD. This finding suggests that functional foods and nutraceuticals may serve as potential sources of LDAI candidates, making them beneficial for managing chronic diseases, such as MAFLD/MASH. Thus, common vegetable extracts, including bean and garlic extracts, collected from the Hokkaido region as a prospective functional food source, have been studied for their potential to inhibit lipid accumulation and have been shown to reduce LD accumulation in cellular experiments. In tests involving hepatocytes, beans extracts have been found to regulate LDA and oxidized lipid formation [24]. The present study focused on the bioactive *Allium* varieties native to Hokkaido, Japan, and their capacity to hinder lipid accumulation in HepG2 cells. Initially, we prepared extracts from nine parts of nine *Allium* species from Hokkaido and evaluated their potential to impede lipid accumulation and oxidation using lipidomics. Finally, metabolomic fingerprinting and rapid dereplication of chemical constituents of identified bioactive extract AL1 were investigated using both NMR and LC-MS techniques (Scheme 1).

## 2. Materials and Methods

### 2.1. Chemical

The chemicals and equipment used in this study are described in the supporting information as previously reported (Supplemental Information: Material and methods). For the TG assay, RIPA Buffer (Nakalai Tesque, Kyoto, Japan), LabAssay Triglyceride (Waco Pure Chemical, Osaka, Japan), and a Pierce BCA Assay Kit (Life Technologies, Carlsbad, CA, USA) were used. EquiSPLASH LIPIDOMIX^®^ quantitative mass spec internal standard (Avanti Polar Lipids, Alabaster, AL, USA) was used as the internal standard for lipidomics. The mobile phases for LC/MS were ammonium acetate (Wako Pure Chemical, Osaka, Japan) and LC-grade methanol (Kanto Chemical, Tokyo, Japan).

### 2.2. Extraction 

The nine *Allium* samples used in this study were collected by Dya Fita Dibwe (D.F.D) and Saki Oba (SO) from the Sapporo market in April 2020 and deposited at the Health Innovation Center of the university’s health science faculty (Table S1, Picture S1). They were listed as follows (code, Scientific name, part used in this study): AL1, *Allium sativum* (bulb), Ninniku; AL2, *Allium victorialis* (leaves), Gyoujaninnniku; AL3, *Allium victorialis* (stem), Gyoujaninnniku; AL4, *Allium schoenoprasum var. foliosum* (leaves), Asatuki; AL5, *Allium schoenoprasum var. foliosum* (stem), Asatuki; AL6, *Allium cepa* (bulb), Kitamanegi; AL7, *Allium cepa* (bulb), Sinntamanegi; AL8, *Allium cepa* (bulb), Sinntamanegi; and AL9, *Allium cepa* (bulb), Sinntamanegi. To identify the bioactive extracts and metabolites derived from food sources, we prepared nine different *Allium* food samples, AL1–AL9. The extraction method was performed as follow: Each of these food items was purchased from a market or supermarket in Sapporo in the spring of 2020 (Table S1, Picture S1). First, 10 g of each sample was ground using a mortar and pestle, and the crushed material was transferred to a beaker. Next, 100 mL of methanol was added to the mixture for one hour. The mixture was sonicated for 30 min, which was repeated twice to obtain the extract. Methanol was removed by decompression and dried to obtain the methanol extract. The biological activities of the extracts were evaluated using a cell viability and lipid droplet accumulation inhibition test.

### 2.3. Cell Viability, Lipid Droplet Accumulation Inhibition Assay and Antioxidant Activity Index

Cell Culture and Cell Viability Assay: HepG2 cells were purchased from the American Type Culture Collection (ATCC) (Manassas, VA, USA). High-glucose DMEM containing 10% heat-inactivated FBS and 1% penicillin–streptomycin was maintained under a humidified atmosphere at 37 °C with 5% CO_2_, as indicated in Supplemental Information: Materials and Methods. HepG2 cells (1.5 × 10^4^/well) in DMEM supplemented with 10% FBS were seeded into a 96-well plate. Cytotoxicity and lipocytotoxicity assays were performed according to the manufacturer’s protocol using CCK-8 (Dojindo Molecular Technologies), as indicated in the Supplemental Information: Materials and Methods. HepG2 cells were purchased from RIKEN BRC cell bank (Ibaraki, Japan). HepG2 cells (1.5 × 104/well) in DMEM supplemented with 10% FBS were seeded into a 96-well plate. The cytotoxicity assay was performed using CCK-8 (Dojindo Molecular Technologies) according to the manufacturer’s protocol. Cell viability was determined using the CCK-8 assay (Dojindo Molecular Technologies, Rockville, MD, USA) according to the manufacturer’s instructions. The cell suspension (100 μL/well) was inoculated in a 96-well plate (Iwaki, Japan), and once monolayers were observed, treatments were added (n = 3 per each treatment). Cells were then pre-incubated with the treatments for 24 h before the assay to determine cell viability. Absorbance was measured at 450 nm using a plate reader (PerkinElmer-ARVO-MX-ID 10533234, Waltham, MA, USA)

LDAI activity was determined using an Oil Red O assay with 24-well plates (n = 4 per treatment) as previously reported. For the LD staining assay, staining was performed as previously described with modifications (Supplemental Information: Materials and Methods). The lipid droplet accumulation inhibition assay and real-time LDAI accumulation were investigated in the present study. Lipid droplet accumulation inhibition (LDAI) activity was determined using an Oil Red assay in a 24-well plate according to the manufacturer’s instructions (n = 4 per each treatment). Oil Red O Staining was carried out as follows: HepG2 cells were cultured and treated in 35 mm dishes, as described above. Oil Red O dye was used for LD staining. Oil Red O was dissolved in isopropanol at 3 mg/mL and diluted with water so that isopropanol became 60%. Oil Red O solution was filtered by a 0.4 μm pore syringe filter. For cellular staining, the culture medium was removed from each dish, and cells were washed and rinsed with 1 × PBS. Cells were then fixed for 20 min in 10% formalin and washed twice using PBS. Then, Oil Red O dye was added to the dishes and incubated for 20 min. Finally, the dye was removed from each dish and the cells. 

The antioxidant activity index (AAI) was evaluated using the methodology outlined by Scherer and Godoy [31], which utilizes the DPPH radical test. The process involved creating a working solution by combining 10 mg DPPH with 100 mL ethanol. A series of extract concentrations ranging from 6.25 to 200 μg/mL were prepared through sequential two-fold dilutions. In a 96-well plate, 100 μL of each diluted sample was combined with an equal volume of the DPPH working solution. After a 30 min incubation period at room temperature in the dark, absorbance measurements were taken at 517 nm. Vitamin C (ascorbic acid) and chlorogenic acid were used as the reference standards. The capacity to neutralize DPPH radicals was determined using the following equation:

%RSA = [(Acontrol − Asample)/Acontrol] × 100

where A represents the absorbance at 517 nm.

Excel was utilized to determine the IC_50_ (the concentration that inhibits 50% of activity) for both extracts and standards, with results visualized using Graphpad Prism v7.0/10.1.2. The antioxidant activity index (AAI) was subsequently computed using the following formula:

AAI = [DPPH] (μg/mL)/IC_50_ (μg/mL)

where [DPPH] represents the final DPPH concentration. We applied the criteria established by Scherer and Godoy [31] to evaluate antioxidant activity: plant extracts’ and compounds’ antioxidant activity was classified as poor when AAI < 0.5, moderate when AAI fell between 0.5 and 1.0, strong when AAI ranged from 1.0 to 2.0, and very strong when AAI exceeded 2.0.

### 2.4. Cell Morphology Alteration: LD Fluorescence Staining Assay and Real-Time LDAI Monitoring

For the LD fluorescence staining assay, staining was performed with modifications based on our previous report as previously indicated in Supplemental Information: Materials and Methods. Real-time oleic acid-induced LD inhibition was performed by using a Nikkon microscope. We performed morphological analysis of HepG2 cells after treatment with control(−OA) and (+OA).

Staining was performed with modifications based on the study by Tsukui et al. [30]. HepG2 cells were seeded in 35 mm glass-bottomed dishes at 4.0 × 10^5^/dish. After 24 h of incubation, OA (0.25 mM) and the samples (150 μg/mL and 300 μg/mL) were added. Then, 24 h after treatment, Oil Red O staining was performed and a solution of Hoechst’s reagent mixed with PBS (Hoechst: PBS = 1:1000) was added. Specifically, after Oil Red O staining, instead of adding 100% IPA, a mixed solution of Hoechst–PBS was added and incubated in the dark for 25 min. The images were captured using a BZ-9000 fluorescence microscope (Keyence Co., Ltd., Osaka, Japan). The filters used were TRITC and DAPI-B (Keyence Co., Ltd., Osaka, Japan).

Real-time LDA and LDAI monitoring: Morphological changes in HepG2 cells and LDs induced by treatment with AL1 was monitored using a Nikon, Hamamatsu Photonics/Ti-E, CCD Camera for 24 h (Supplemental Information: Video S1).

### 2.5. TG Assay

HepG2 cells were seeded in 12-well plates at 3.0 × 10^5^/well; 24 h later, OA (0.25 mM) and the samples (150 μg/mL and/or 300 μg/mL) were added and incubated for 24 h, after which triacylglycerol and protein levels were measured. For triacylglycerol and protein extraction, Poudel’s method and the protocol of the ATTO EzRIPA Lysis Kit were used as references. The medium was discarded, 150 μL of trypsin was added, the cells were incubated for 15 min, and the cells were collected. After centrifugation (500× *g*, 4 °C, 5 min), the supernatant was discarded, and 400 μL of PBS was added to wash the cells. After centrifugation, the supernatant was discarded and the cells were washed with PBS and centrifuged. After removing the supernatant, 500 μL of RIPA buffer was added; the mixture was incubated on ice for 15 min and mixed by inverting every 1–2 min. Next, centrifugation at 14,000× *g* was carried out, and the supernatant was transferred to a new tube and stored at −80 °C. Subsequent triacylglycerol and protein measurements were performed according to the manufacturer’s protocol provided in the kit. The plate reader used was an xMark (Bio-Rad, Hercules, CA, USA). The triacylglycerol results were corrected for proteins. In the TG assay, the total amount of triacylglycerol was determined by correcting the concentration obtained using the TG kit to the total amount of protein. 

### 2.6. Lipidomic Analysis of Neutral Lipids: Analysis of Accumulation of Triacylglycerol Species by LC-MS/MS

HepG2 cells were seeded at 2.0 × 10^5^ cells/dish in 35 mm culture dishes and incubated for 24 h. Subsequently, OA and the samples were added to the cells. The OA concentration was 0.25 mM, and two sample concentrations, 150 μg/mL and 300 μg/mL, were selected. The next day, lipids were extracted from the cells and analyzed using LC-MS. The extraction procedure was as follows: First, the medium was removed and the cells were washed twice with 300 μL of PBS. Then, 300 μL of trypsin was added and incubated for 10 min at 37 °C and 5% CO2. Next, 300 μL of DMEM was dispensed and the cells were collected in Eppendorf tubes. Both tubes (550 μL) were centrifuged for 1 min at 13,000 rpm and 4 °C. The supernatant was removed from both tubes and 400 μL of chloroform, 150 μL of methanol, and 10 μL of the internal standard were added to one tube. The tubes were vortexed at 3500 rpm for 10 min and centrifuged at 15,000 rpm at 4 °C for 15 min. The supernatant was transferred to a separate tube, and 400 μL of chloroform and 150 μL of methanol were added to the cell mass, vortexed, and centrifuged in the same manner. The supernatant was then transferred to the same tube. After evaporating the solvent, 100 μL of methanol was dispensed for LC, vortexed at 3500 rpm for 3 min, and centrifuged at 15,000 rpm at 4 °C for 10 min.

LC-MS (positive mode) was used to analyze the supernatant. A Shimadzu Prominence HPLC system (Shimadzu Corporation, Kyoto, Japan), LTQ Orbitrap mass spectrometer (Thermo Fisher Scientific Inc., San Jose, CA, USA), and electrospray ionization (ESI) were used in the analysis. Electrospray ionization (ESI) was used for ionization. The samples were separated on an Atlantis T3 C18 column (2.1 × 150 mm, 3 μm, Waters, Milford, MA, USA) at a flow rate of 200 μL/min, and the column temperature was maintained at 40 °C. LC gradient elution was performed using a mobile phase consisting of a 10 mM ammonium acetate solution, isopropanol, and methanol. The measurements were carried out in positive mode: the volTGe of the MS capillary was 4.04 kV, the flow rate of the sheath gas (nitrogen) was 50 psi, and the auxiliary gas (nitrogen) was 20 psi. High-resolution MS data were obtained in the scan range *m*/*z* 150–1200. The molecular species of the lipids analyzed were TAG and their oxidized lipids. The results have been corrected for the number of cells.

The crude extracts (200 μM) were added to HepG2 cells that had been preloaded with oleic acid and then incubated at 37 °C for 24 h, following a previously established protocol [22,25,26]. After centrifugation, the samples were analyzed using an LC/MS Orbitrap. A Shimadzu Prominence UHPLC system with a binary solvent delivery system and standard autosampler was employed for chromatographic separation. The Atlantis^®^ T3 column (2.1 × 150 mm, 3 μm, Waters) was used for separation, with a flow rate of 200 μL/min. The mobile phase consisted of a 10 mM ammonium acetate solution (A) in isopropanol (B) and methanol (C). In positive mode, the percentages of solvents B and C were as follows: 0–1 min, 6% B and 90% C; 1–10 min, 83% B and 15% C; 10–19 min, 83% B and 15% C; 19–19.5 min, 6% B and 90% C; and 19.5–22 min, 6% B and 90% C. The injection volume was 10 μL and the column temperature was maintained at 40 °C. 

### 2.7. Metabolite Profile of AL1 Extract

LC-grade methanol (Kanto Chemical, Tokyo, Japan) was used as the mobile phase. The column utilized was CAPCELL PAK C18 UG120Å 5 μm (Osaka Soda, Osaka, Japan), while Methanol-d4 and DMSO-d6 (Sigma-Aldrich, St. Louis, MO, USA) were used as solvents for the NMR analysis.

For HPLC analysis, AL1 and AL6 extracts were dissolved in LC-grade methanol to a concentration of 100 mg/mL and filtered through a 0.45 μm, 13 mm syringe filter from Hawac Scientific (Xi’an, China). The samples were analyzed using a Shimadzu Prominence HPLC system (Shimadzu Corporation, Kyoto, Japan) with a column for separation (CAPCELL PAK C18 UG120 Å, 25 × 0.1 cm, 5 μm particle size). The mobile phases consisted of Milli-Q water for A and HPLC-grade methanol for B at a 5.0 mL/min flow rate. The solvent proportions were 0–1 min, 20% B; 1–20 min, 100% B; and 20–23 min, 100% B, and the post-run time was set at 2 min. Chromatograms were recorded at 200, 275, and 343 nm.

Nuclear magnetic resonance (NMR) measurements were also performed. The extracts were dissolved in methanol-d4 at a concentration of 10 mg/mL and analyzed via 400 MHz JNM-ECX400P (JEOL, Tokyo, Japan) based on ^1^H, ^13^C, and Dept (135 and 90) measurements. The spectra were processed using JOEL v6.3.0 software, and chemical shift values are expressed in ppm.

Liquid chromatography/mass spectrometry (LC/MS) analysis was also performed. The extracts were dissolved in methanol to obtain a concentration of 100 μg/mL for LC-MS analysis. The samples were filtered and analyzed using an LC/MS system that combined a Shimadzu Prominence HPLC system (Shimadzu Corporation, Kyoto, Japan) and an LTQ Orbitrap mass spectrometer (Thermo Fisher Scientific Inc., San Jose, CA, USA). The samples were separated using an Atlantis T3 C18 column (2.1 × 150 mm, 3 μm, Waters, Milford, MA, USA) at a flow rate of 200 μL/min. The column and sample tray were maintained at 40 and 4 °C, respectively. The measurements were performed in the positive mode, and the mobile phases of the LC were 10 mM ammonium acetate solution, isopropanol, and methanol. The MS/MS data were acquired in ion-trap mode and operated in data-dependent mode.

A Shimadzu LC system (Shimadzu Corporation, Kyoto, Japan) linked to an LTQ Orbitrap XL (Thermo Fisher Scientific Inc., San Jose, CA, USA) mass spectrometer was used to examine the methanolic extracts of all nine samples. An Atlantis T3 C18 column (2.1 × 150 mm, 3 μm, Waters, Milford, MA, USA) was used for sample separation at a flow rate of 200 μL/min, with the column and sample tray kept at 40 °C and 4 °C, respectively. Chromatographic separation was achieved using a 10 μL injection volume and a gradient elution program involving 10 mM ammonium acetate solution, isopropanol, and methanol. Both the HREIMS and HREIMS/MS analyses used a heated electrospray ionization (HESI) source with positive ionization modes. MS data were acquired in the ESI-positive mode. High-resolution masses were obtained using a Fourier transform (FT) full scan range of *m*/*z* 50–500 for the MS1 spectra. Low-resolution MS/MS spectra were acquired through data-dependent acquisition using collision-induced dissociation (CID) in ion-trap mode. The resulting raw data were processed using Xcalibur 2.2 (Thermo Fisher Scientific Inc., San Jose, CA, USA), applying a 5.0 ppm mass tolerance. MZmine 2 (http://mzmine.github.io accessed on 7 March 2022) was used for HREIMS feature detection. Metabolites identified in Allium had RT and MS values comparable to those of standards. The analysis of the results and metabolite identification were conducted using the vendor software Xcalibur.

## 3. Results and Discussion

### 3.1. Cell Viability

**Figure 1:**
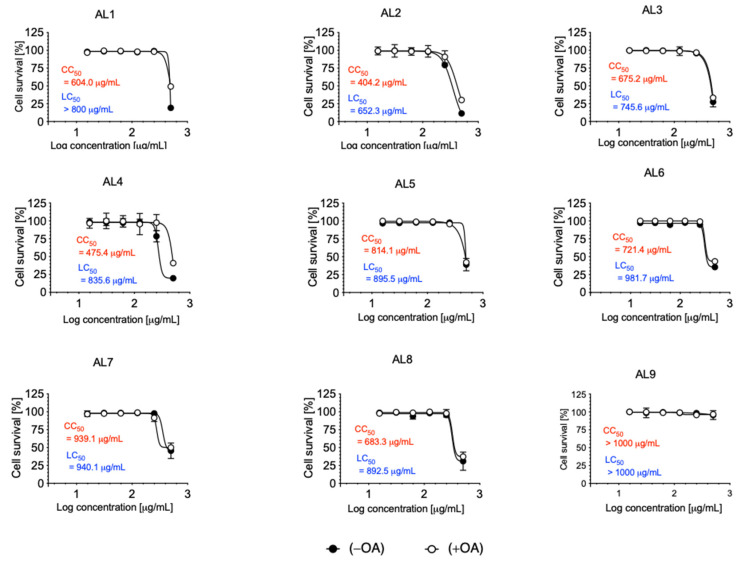
Cytotoxicity and lipocytotoxicity of AL1–9 in HepG2 cells. The cytotoxicity (CC_50_ value in red) was expressed as the concentration at which 50% of cells died in DMEM condition without fatty acid (−OA). The lipocytotoxicity (LC_50_ value in blue) was expressed as the concentration at which 50% of cells died, especially in DMEM condition loaded with fatty acid (+OA).

Cytotoxicity and lipocytotoxicity were measured using CCK-8, and CC_50_ and LC_50_ were calculated. The concentrations added ranged from 500 to 15.625 μg/mL. In all extracts, the CC_50_ and LC_50_ were higher than 400 μg/mL (Figure 1). Our previous results indicate that nutraceuticals and functional foods are effective options for managing metabolic disorders through the use of candidate LDAIs and antioxidants. These results are supported by numerous studies suggesting that a significant number of nutraceuticals and functional foods are derived from natural plant sources or combinations of natural products. 

Thus, the current study further investigated LDAI and oxLDAI under nontoxic conditions, particularly in relation to the nonlipocytotoxic loads of oleic acid (OA) and linoleic acid (LA).

### 3.2. Lipid Droplet Accumulation Inhibition Activity of Selected Allium Extracts

**Figure 2:**
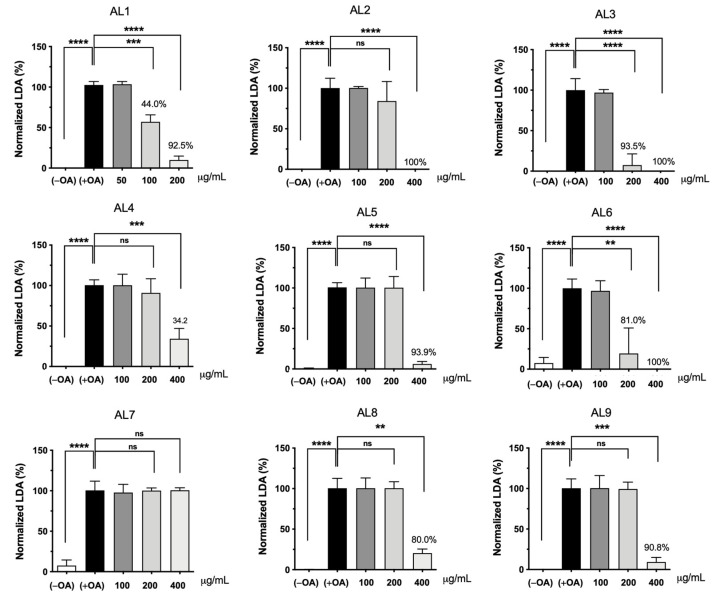
LDAI activity of selected bean extracts on OA-loaded HepG2 cells. Graph showing the mean values of the LDAI (four replications). **** *p* < 0.0001, *** *p* < 0.001, ** *p* < 0.01, one-way analysis of variance (ANOVA) with Tukey’s multiple comparisons test when compared with the untreated control.

The accumulation of intracellular lipid droplets (LDs) increases in a concentration-dependent manner under oleic acid (OA) conditions. When HepG2 cells were incubated for 24 h under OA loading (0.1 to 0.5 mM in the nontoxic range), LD accumulation increased predominantly from 0.25 mM. Real-time observation of this phenomenon showed that intracellular LD levels increased while cells remained alive [18,19,22,26,27,28,29]. Based on these results, the nine selected Allium extracts were simultaneously co-treated with 0.25 mM OA and incubated for 24 h. The results calculated after measuring absorbance are shown in Figure 2. Interestingly, AL1, AL3, and AL6 showed a significant decrease in absorbance even at 200 μg/mL. Among the nine samples, AL1, AL3, and AL6 demonstrated a substantial decrease in absorbance, even at 200 μg/mL, with LDAI values of 92.5, 93.5, and 81.0%, respectively, compared to the other extracts. Thus, AL-1 extract was selected to investigate alterations in cell morphology induced by AL1 and TG assay.

### 3.3. Antioxidant Activity Index of AL1–9

The significance of free radicals in biological and medical research has steadily increased. These molecules are generated through various internal and external processes, with mitochondria serving as the primary source of cellular reactive oxygen species (ROS). Excessive production of free radicals can inflict damage to essential macromolecules, including nucleic acids, proteins, and lipids, resulting in tissue deterioration associated with numerous chronic and degenerative conditions. Free radicals induce oxidative damage in biomolecules. This damage causes atherosclerosis, aging, cancer, and several other diseases [32]. Free radicals participate in lipid peroxidation. Antioxidants play a vital role in protecting the body from free radicals. This study examined the antioxidant activity index of nine selected Allium samples (AL1–9) and two known antioxidant (KA) compounds, chlorogenic acid (KA1) and ascorbic acid (Vitamin C, KA2). It also explored the potential benefits of antioxidant dietary supplementation and addressed unresolved issues concerning the use of antioxidant supplements in disease prevention and treatment via the inhibition of LDA and oxLDA in fatty acid-loaded cell lines. The antioxidant activity, cell viability, and LDAI of the selected Allium AL1–9 were evaluated. The antioxidant activity index (AAI) was evaluated using the following criteria: IC_50_ ≥ 2.0, very strong; in the range of 1.0–2.0, strong; 0.5–0.1, moderate; and IC_50_ < 0.5, poor. No cytotoxicity or lipocytotoxicity was observed for any of the tested samples. Significant LDAI was found for AL1, AL3, and AL6 under LA compared to other samples, while the DPPH assay revealed antioxidant activity, with the strongest antioxidant activity index for AL4, followed by AL3 and AL6, and finally AL1, AL5, and AL7. A poor antioxidant activity index was observed for both AL8 and AL9. Chlorogenic acid (KA1) and ascorbic acid (Vitamin C, KA2) were used as known antioxidants, both of which exhibited very strong AAI and showed no cytotoxicity and no lipocytotoxicity in hepatocytes; however, only KA1 showed a moderate LDAI, while ascorbic acid did not show any inhibition. These results suggested that not all direct antioxidants can inhibit LDA/oxLDA, which is associated with the regulation of Keap1-NRF2. AL1, AL1, AL3, and AL6 showed significant LDAI and AAI, necessitating a more detailed lipidomic analysis of fluctuations in intracellular neutral lipids induced by LA targeting TAG and their oxidative molecular species.

### 3.4. Cell Morphology Alteration Induced by Allium Extract AL1 

**Figure 3:**
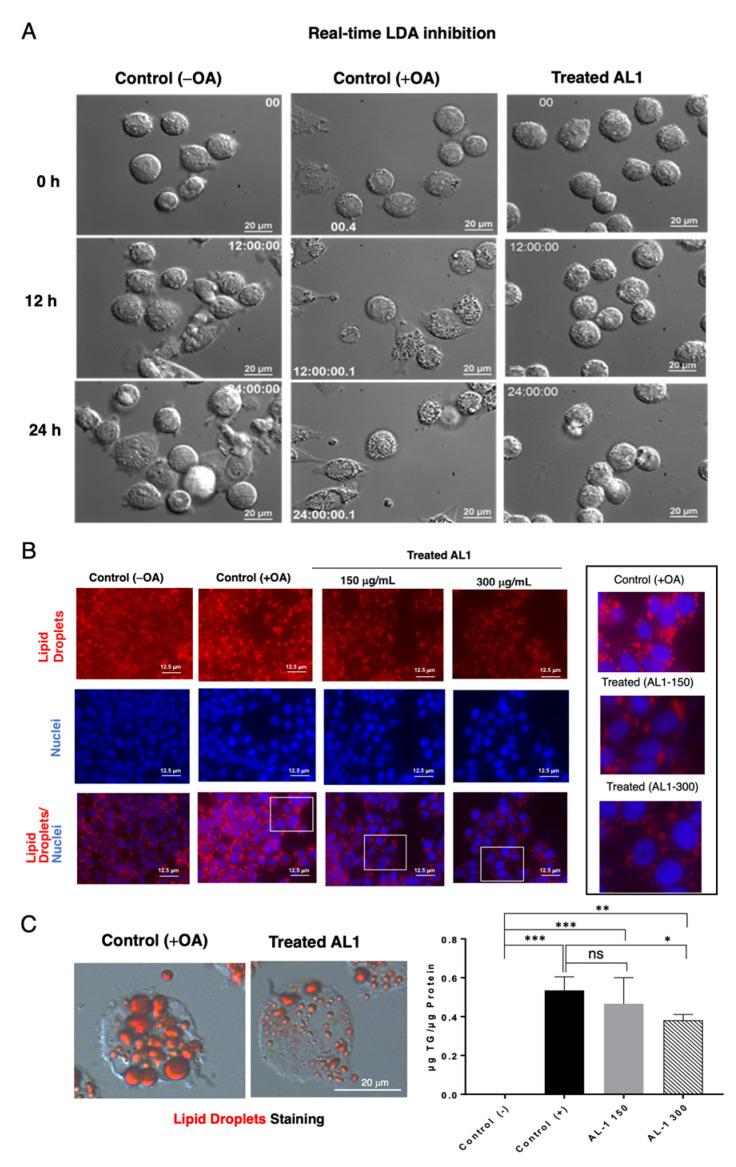
(**A**) Capture of the LDA and LDAI real-time images on HepG2 cells. (**B**) Fluorescence staining of cells treated with AL1. (**C**) Oil Red O staining after 24 h treatment with AL1 and total triacylglycerol amount with AL1. Graph showing the mean values of the LDA and LDAI (six replications). *** *p* < 0.001, ** *p* < 0.01, * *p* < 0.05; one-way analysis of variance (ANOVA) with Tukey’s multiple comparisons test when compared with the untreated control (+OA) group. ns, not significant; TAG, triacylglycerol; OA, oleic acid.

We measured the real-time LDA and inhibition of AL1 24 h after cell seeding, after OA and the AL1 sample were added, and real-time images were recorded. At 12 h after OA addition, lipid droplet accumulation was observed in the control group. In contrast, in the treated cells, lipid droplet accumulation was suppressed after 12 h of treatment. In this study, real-time LDA inhibition of AL1 was performed (Supplementary Information Video S1). Oil Red O staining was performed after real-time imaging. The size and number of lipid droplets were smaller in cells containing the extract than in those containing lipids alone (Figure 3). For double staining of hepatocytes, Oil Red O staining was used to stain the LDs and Hoechst staining was used to stain the nuclei. Red color represents lipid droplets, blue color represents the cell nucleus, and the lower part is the merged image. A concentration-dependent inhibitory effect was apparent when the sample concentration was 300 µg/mL, as evidenced by the smaller number and size of lipid droplets compared to those at 150 µg/mL. This suggests that AL1 inhibited lipid droplet accumulation in a concentration-dependent manner during cell fatty acid loading, as depicted in Figure 3A–C.

### 3.5. Level of TG After AL1 Treatment

The level of triacylglycerol was assessed by correcting the concentration obtained using the TG kit against the total amount of protein present. With the addition of OA, triacylglycerol levels in cells increased compared with those in the absence of OA. Furthermore, when 150 μg/mL extract was added, the amount of triacylglycerol decreased by approximately 13%, and when 300 μg/mL extract was added, the amount of triacylglycerol decreased by 30%. These results indicated a concentration-dependent decrease in the amount of triacylglycerol (Figure 3C).

### 3.6. Quantification of the Effect on Inhibition of TAG and TGOOH Species Accumulation Under LDA and oxLDA Conditions

The accumulation of lipid droplets, primarily in the form of triacylglycerols (TAGs), in the liver is a characteristic feature of MAFLD. Oxidative stress, as indicated by the presence of lipid oxidation byproducts, has been implicated in the progression of MAFLD, including MASH and related disorders [10,11,18,23,24,25,26,33,34]. This study aimed to comprehensively analyze the changes in TAG and oxidized TGOOH species in HepG2 cells treated with AL1, a bioactive LDAI extract, after exposure to two lipid-loading conditions, OA and linoleic acid (LA). Lipidomic analysis of neutral lipids, TAG, and oxidized TGOOH species was performed using LC-MS. Lipid droplets were extracted from the cells and lipidomic experiments were conducted under two different conditions (OA and LA) in HepG2 cells. 

The results were corrected using the number of cells and internal standards. Under OA conditions, 51 triacylglycerol molecular species were detected in control cells and 53 triacylglycerol molecular species were detected in the extract of control cells treated with LA (Tables S2 and S3). In addition to triacylglycerol molecules, hydroxyperoxides of triacylglycerol were detected in the present study at three different oxidation levels, TG(OOH) n = 3 (Supplemental Information: Materials and Methods, Tables S4 and S5). Lipid peroxides are significantly increased in MASH liver models and are believed to contribute to the progression of MASH by disrupting the structure and function of biomembranes composed of lipids. To thoroughly examine the effects of AL1 on lipid hydroperoxides, LC-MS/MS analysis was used to assess the changes in TAG-neutral lipids and TGOOH-oxidized species in HepG2 cells under OA and LA conditions. This was performed to investigate the impact of AL1 on the specific components of lipid hydroperoxidation using our previously reported oxidative lipidomics approach [25].

**MS technology—MS/MS for the detection of oxidized molecular neutral lipid species.** In our previous report, the aforementioned explanation highlighted the increasing trend towards advanced MS approaches in support of conventional imaging methods. A key aspect of lipid oxidation is the formation of lipid hydroperoxides, which are composed of an additional hydroperoxyl group and undergo the rearrangement of their C=C double bonds. The production of hydroperoxides (−OOH) is influenced by the source of oxidation, including radical oxidation (e.g., thermal oxidation and auto-oxidation), enzymatic oxidation (e.g., lipoxygenase), and singlet oxygen oxidation (e.g., photooxidation and inflammation). The specific characteristics of the hydroperoxides produced depend on the oxidation process used. Subsequent reactions initiate a chain reaction that continues without interruption [12,27,28]. Our objective was to bridge the gap in traditional approaches to oxidized lipid analysis in LD lipidomes by focusing on advanced methods and strategies for oxidative lipidomics. Generally, the examination of oxidized lipids relies on indirect detection of lipid oxidation byproducts, which can be quantified using colorimetric tests, immunoassays, or electron spin resonance spectroscopy. The following methods are utilized: thiobarbituric acid-reactive substances (TBARSs) and malondialdehyde (MDA) tests, ferrous oxidation in xylenol orange (FOX), and gated diene tests. Although these methods, including recent fluorescence techniques, are typically used to gauge the degree of lipid oxidation, they are not always reliable for providing detailed analyses of changes in lipid species [12,27,28,29,30]. To overcome this limitation, the detection of oxidized molecular lipid species using MS/MS-based approaches has shown great promise [12,27,28,29,30]. 

MS/MS-based detection of oxidized molecular lipid species using MS technology effectively tackles the challenges of sensitivity, specificity, accuracy, and dynamic range, and facilitates high-throughput analysis. In contrast, other methods may necessitate selection and customization to assess lipid oxidation products. This is particularly advantageous for identifying oxidized lipids present in intricate mixtures of varying concentrations and structural complexities. The MS-based detection of oxidized lipids is an attractive solution [28]. Currently, LC-MS techniques, particularly LC-MS/MS, are widely used for oxidative lipidomic applications owing to their effectiveness [31]. This is because oxidized lipids have additional oxygen atoms attached to their molecules, such as peroxyl, hydroperoxyl, or epoxy groups, or the deletion of C=C double bonds. These oxidized lipid molecules can be identified by using MS, particularly high-resolution MS. Moreover, MS/MS can be used to confirm their structures, including the presence of oxygen, the position of oxidation, and specific fragmentation from precursor ions to product ions.

**Figure 4:**
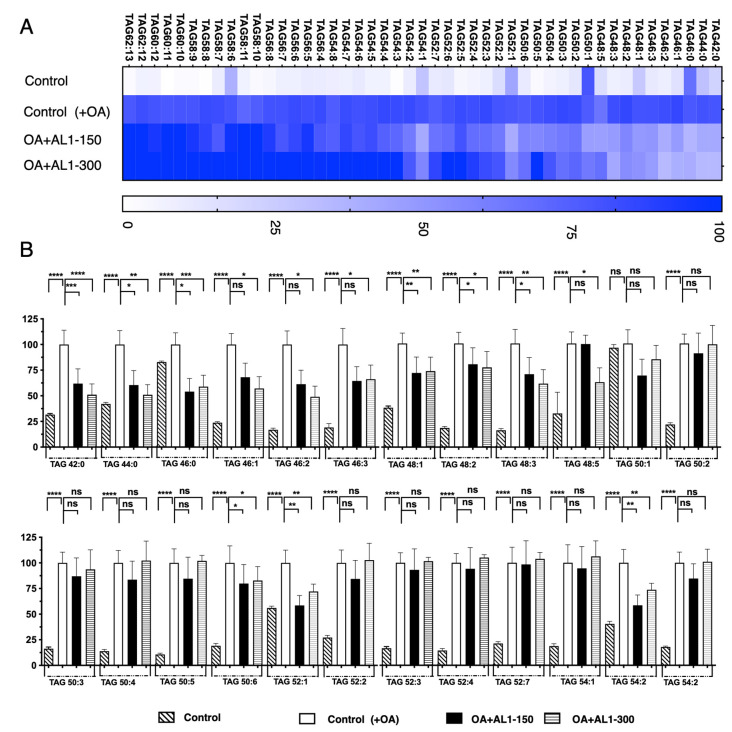
(**A**) Heat map of accumulated and inhibited TAG species in cells under OA. (**B**) Quantification of fluctuation of accumulated TAG species under OA treatment. Analysis of triacylglycerol molecular species under OA treatment with AL1. Graph showing the mean values of the LDA and LDAI (six replications). **** *p* < 0.0001, *** *p* < 0.001, ** *p* < 0.01, * *p* < 0.05; one-way analysis of variance (ANOVA) with Tukey’s multiple comparisons test when compared with the untreated control (+OA) group. ns, not significant; TAG, triacylglycerol; OA, oleic acid.

**Analysis of TAG and TGOOH species accumulation and inhibition in linoleic acid-loaded hepatocytes.** OA-treated HepG2 cells showed a significant increase in LDA, and significant LDAI was observed for AL1, AL3, and AL6 samples, as shown in Figure 4. The bioactive extract samples AL1, AL3, and AL6 inhibited LDA in a concentration-dependent manner in HepG2 cells treated with oleic acid. In this study, we analyzed the effects of LDAI bioactive *Allium* extract AL1 on TAG species accumulation in hepatocytes using LC-MS. In this study, AL1 (0.25 mM and 0.150 mM) significantly inhibited LDA in HepG2 cells loaded with 0.25 mM OA at 100% and 56%, respectively. The profiles of neutral lipid TAG and TG(OOH) n = 3 species in cells incubated for 24 h were analyzed using Orbitrap LC-MS. All the accumulated TAG and TGOOH species induced by OA were detected (Supplemental Information: Tables S2 and S3). In this study, we used lipidomic analysis to assess the effects of OA loading on control cells. Our results showed that 51 triacylglycerol molecular species were present, of which 25 were reduced upon treatment with AL1 (Supplemental Information: Tables S4 and S5). Triacylglycerol hydroperoxides were also detected in addition to triacylglycerol molecules. A heat map was used to display the rate of accumulation in each group of cells, where the accumulation in the control cells treated with fatty acids was set to 100%. The darker the color, the higher the accumulation. The heat map revealed that OA-treated cells contained numerous molecular species of triacylglycerols that accumulated in the cells. AL1 treatment significantly suppressed nine TAG molecular species at 150 μg/mL (TAGs 42:0, 44:0, 46:0, 48:1, 48:2, 48:3, 50:6, 52:1, and 54:2) and four additional TAG molecular species at 300 μg/mL (TAGs 46:1, 46:2, 46:3, and 48:5) (Table 1). Using a lipidomic approach, we quantified triacylglycerol molecular species accumulated and inhibited under FFA-loaded conditions. Our findings showed that TAG42:0, TAG44:0, and TAG48:5, showed the greatest reduction in intracellular accumulation, with a reduction of approximately 40%. The accumulation of TAGs 42:0, 44:0, and 48:5 was reduced by 25–49%, while the inhibition of TAGs 46:0, 46:1, 46:2, 46:3, 48:1, 48:2, 48:3, 50:6, 52:1, and 54:2 was in the range of 1–25%. These results confirmed that AL-1 treatment reduced the accumulation of triacylglycerol molecular species and triacylglycerol hydroperoxide (Figure 4A,B).

**Figure 5:**
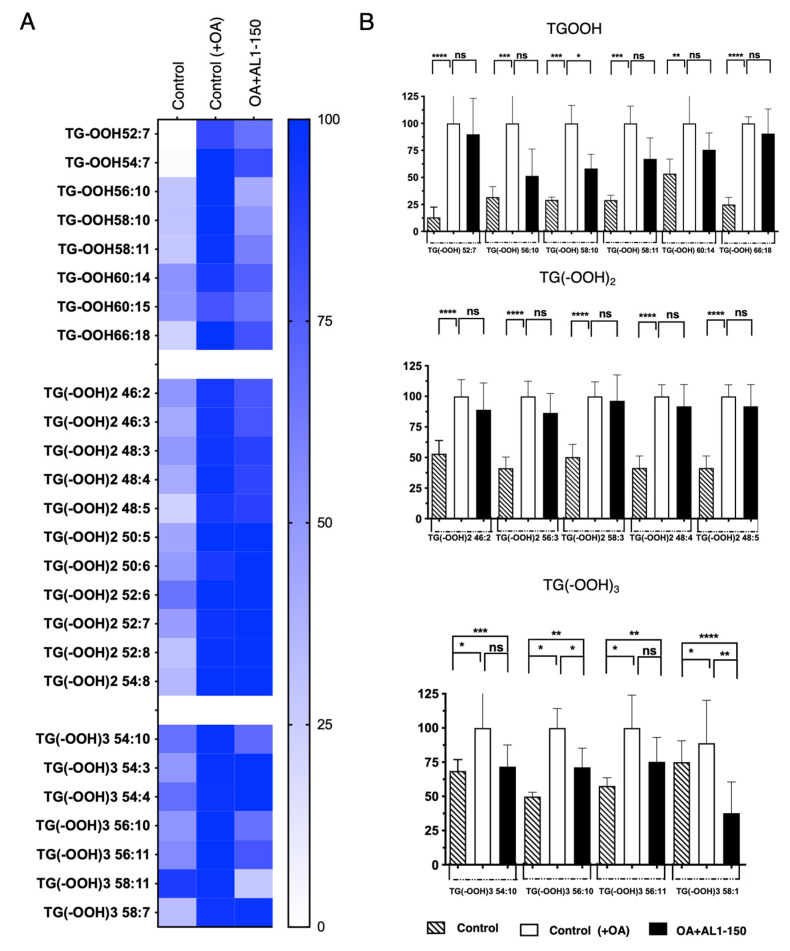
(**A**) Heat map of accumulated and inhibited TGOOH species in cells under OA. (**B**) Quantification of fluctuation of accumulated TGOOH species under OA treatment. Analysis of TAG molecular species under OA treatment with AL1. Graph showing the mean values of the LDA and LDAI (six replications). **** *p* < 0.0001, *** *p* < 0.001, ** *p* < 0.01, * *p* < 0.05; one-way analysis of variance (ANOVA) with Tukey’s multiple comparisons test, when compared with the untreated control (+OA) group. ns, not significant; TGOOH, triacylglycerol hydroperoxide; OA, oleic acid.

Under OA-loaded conditions, as shown in Figure 5, Table 2, and Table S5, oxidized TGOOH species were detected as follows: 8 TGOOH, 11 TG(−OOH)_2_, and 6 TG(−OOH)_3_. Among these, zero TG(−OOH)_2_ species were suppressed by AL1 treatment at 150 mg/mL, whereas significant inhibition was observed for one TGOOH, TG(−OOH) 58:10 and two TG(−OOH)_3_ species, TG(−OOH)_3_ 52:3 and TG(−OOH)_3_ 52:5, suggesting that AL1 may contain potential bioactive LDAI candidates for reducing the accumulation of oxidized molecular lipids in LDs.

**Quantification of TAG and TGOOH species accumulation and inhibition in linoleic acid-loaded hepatocytes.** Our previous study revealed that LA causes not only LDA but also excessive oxLD accumulation (oxLDA) in hepatocytes [30]. Our group developed a new imaging method under FA-loaded conditions to assess the degree of oxidation using fluorescence microscopy to characterize the size, quantity, and oxidation of lipid droplets in HepG2 cells. This method was compared to conventional approaches. Fluorescent reagents were used to stain neutral lipids and lipid peroxide of LDs induced by PA, OA, or LA and the results showed that the amount and degree of LD oxidation increased in a time-dependent manner for each fatty acid, suggesting the levels of intracellular triglyceride hydroperoxide. Long-chain lipids (LDs) subjected to lipid peroxidation (LP) induced by LA without 2,2′-azobis(2-amidinopropane) dihydrochloride (AAPH) demonstrated the most significant level of oxidation when compared to PA and OA, particularly in LDs with a large surface area (≥3 μm2, oxLD/LD = 52.3 ± 21.7%). Under these conditions, the efficacy of two antioxidants derived from food sources, chlorogenic acid and DHMBA, was examined, and both were found to significantly enhance the LD characteristics. In addition, chlorogenic acid decreased the number of large lipid droplets (LDs) by 74.0–87.6% in a dose-dependent manner, presenting a new strategy to evaluate the effects of dietary antioxidants on LD characteristics. Our group have developed a novel fluorescence-based method to characterize the number, size distribution, and degree of oxidation of LDs in human hepatocytes. Two fluorescent probes were used to assess intact and oxidized LDs (oxLDs). TG hydroperoxides (TG-OOH) were quantified using liquid chromatography–mass spectrometry (LC-MS)/MS and compared with the suggested method to ascertain changes in molecular species and gain comprehensive information about the degree of oxidation. The following information outlines the contrasting effects of two antioxidants, chlorogenic acid and DHMBA, on the modification of intracellular lipid species within LDs [27].

Based on our previous discovery, a similar experiment was conducted that replicated the one in HepG2 cells with OA, but with LA replacing the fatty acids. The purpose of this experiment was to analyze in detail the changes in the fluctuation of accumulated TAG and TGOOH induced by *Allium* AL1-LDAI extract using LC-MS/MS. Orbitrap LC-MS detected accumulated TAG and TGOOH species induced by LA. 

**Figure 6:**
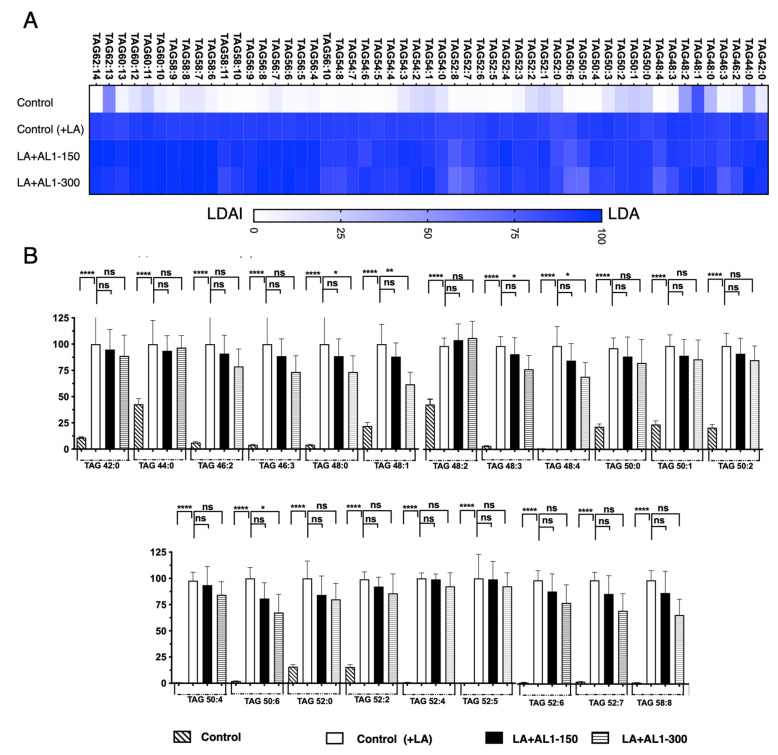
(**A**) Quantification of the fluctuation of accumulated TAG species under LA treatment. (**B**) Analysis of triacylglycerol molecular species under OA treatment with AL1. Graph showing the mean values of the LDA and LDAI (six replications). **** *p* < 0.0001, ** *p* < 0.01, * *p* < 0.05; one-way analysis of variance (ANOVA) with Tukey’s multiple comparisons test when compared with the untreated control (+LA) group. ns, not significant; TAG, triacylglycerol; LA, linoleic acid.

**Figure 7:**
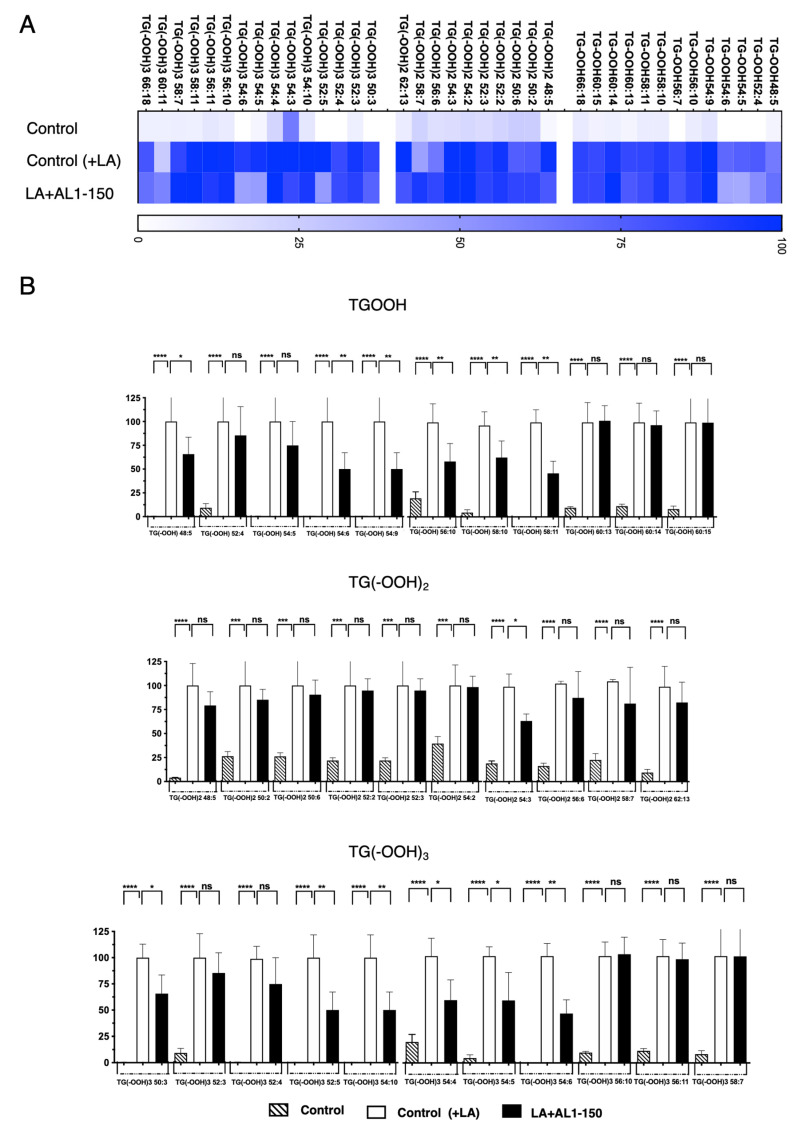
Quantification of fluctuation of accumulated TGOOH species under LA treatment. (**A**) Analysis of triacylglycerol molecular species under LA treatment with heat map and (**B**) accumulation and inhibition of TG(OOH) n = 3 under LA condition. Graph showing the mean values of the LDA and LDAI (six replications). **** *p* < 0.0001, *** *p* < 0.001, ** *p* < 0.01, * *p* < 0.05; one-way analysis of variance (ANOVA) with Tukey’s multiple comparisons test when compared with the untreated control (+LA) group. ns: not significant; TGOOH, triacylglycerol hydroperoxide; LA, linoleic acid.

This advanced lipidomic approach was used to quantify the accumulation and inhibition of intracellular triacylglycerol (TAG) and its oxidized species, TAG hydroperoxide (TGOOH), in hepatocytes under fatty acid-loading conditions. The results show that the antioxidant *allium* sample AL1 is a potential candidate for regulating TAG and TGOOH accumulation in fatty acid-induced lipid droplets (LDs). This study suggests that *allium*-based foods inhibit LD formation by decreasing intracellular lipids and lipid hydroperoxides in the hepatocytes. Thus, we extended the investigation to the bioactive *Allium* AL1. The fluctuation of the TGOOH species was analyzed under LA conditions. When hepatocytes were treated with AL1, 53 accumulated molecular species of triacylglycerols were detected using LC-MS/MS (Figure 6, Table S4). However, upon treatment with AL1, 51 triacylglycerol molecular species were found to be suppressed (Figure 6, Table S5). Triacylglycerol hydroperoxide was also detected. Figure 6 illustrates the triacylglycerol molecular species that hinder the accumulation of AL1. TAG52:4, TAG54:5, and TAG54:6 were the main triacylglycerol molecular species, while minor species, such as TAG42:0, TAG44:0, and TAG54:0, were also detected. A heat map was created for further analysis (Figure 6). To investigate the inhibition of the accumulated molecular species in more detail, omics quantification was performed (Figure 6). The heat map shows the accumulation rate for each cell group, with darker colors indicating higher levels of accumulation. The heat map revealed that OA-treated cells contained numerous molecular species of triacylglycerols that accumulated in the cells. Zero inhibition of TAG molecular species was observed at 150 μg/mL and six TAG molecular species were significantly inhibited at 300 μg/mL (TAGs 48:3, 48:0, 48:1, 48:3, 48:4, and 50:6) (Table 3). Using a lipidomic approach, we quantified the accumulation and inhibition of triacylglycerol molecular species under LA-loaded conditions. Our findings showed that TAGs 48:1, 46:3, and 48:0 have the greatest reduction in intracellular accumulation, with a reduction of approximately 40%. For TAGs 48:1, 46:3, and 48:0, accumulation was reduced in the range between 25 and 49%, while the inhibition of TAGs 48:3, 48:4, and 50:6 was in the range of 1–25%. This study was extended to analyze of the fluctuation of the TGOOH species under LA conditions after treatment with bioactive *Allium* AL1. To further investigate the hydroperoxide content of the TG molecular species, a heat map was created (Figure 7). 

The heat map shows the accumulation rate for each cell group, with darker colors indicating a greater accumulation. MS/MS-based detection of oxidized molecular lipid species enabled us to clearly observe fluctuations induced by AL1 LDAI bioactive food extracts in individual minor and major accumulated lipid molecular species of TAG and TGOOH. The results reveal that approximately 38 accumulated molecular TG(OOH)n species were detected under LA conditions. We detected 13 TGOOH, 10 TG(−OOH)_2_, and 15 TG(−OOH)_3_ species under LA loading. Among them, six TGOOH species (TGOOHs, 48:5; 54:6; 54:9; 56:10; 58:10; and 58:11), one TG(−OOH)_2_ species (TG(−OOH)_2_:54:3), and six TG(−OOH)_3_ species (TG(−OOH)_3_, 50:3; 52:5; 54:10; 54:4; 54:5; and 54:6) were significantly suppressed by AL1. The most inhibited lipid hydroperoxides were the following six TG(−OOH)_3_ species: 50:3; 52:5; 54:10; 54:4; 54:5; and 54:6. This method allowed for sensitive and accurate quantification, with clear discrimination between the two OA- and LA-loading conditions. By extending these results to various FFAs and their combinations, we may gain more insight into the mechanism and diagnosis of oxidative stress via LOOH molecular species. This could potentially expand our understanding of the prevention and discovery of drug targets for MAFLD/MASH and related metabolic disorders.

### 3.7. Metabolite Fingerprinting and Rapid Dereplication of Key Compounds in Bioactive AL1 Extract

Metabolomic analysis enables rapid determination of intricate mixtures of organic compounds, including vegetal extracts. In this field, both liquid and gas chromatography combined with mass spectrometry (LC-MS and GC-MS) and nuclear magnetic resonance (NMR) spectroscopy have gained prominence for the direct identification of natural products (NPs) in complex herbal matrices. For the past decade, automated dereplication processes based on ^13^C-NMR have emerged to efficiently identify major compounds in mixtures using moderate field instruments (400 MHz), freely available automation procedures, and dedicated software. While higher sensitivity is a significant advantage of MS, ^13^C-NMR and Dept (135 and 90) are highly suitable for the discrimination of diastereomers and overlapping metabolites in ^1^H-NMR. This study combined LC-MS/MS techniques and 1D-NMR [^1^H, ^13^C, and Dept (135 and 90)] for profiling and rapid dereplication of potential chemicals. 

**Figure 8:**
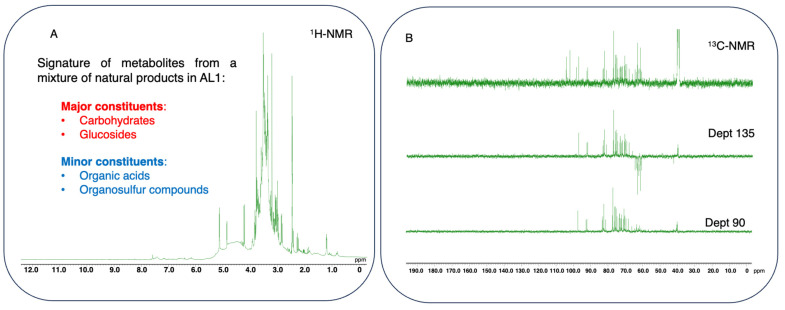
Fingerprinting and signature of potential metabolites from a mixture of natural products in AL1 food extract using 1D-NMR spectra. (**A**) ^1^H-NMR profile of AL-1 in DMSO with *δ*_H_ ranging from 0 to 12.0 ppm. (**B**) ^13^C-NMR and Dept (135 and 90) spectra of AL1 in DMSO with *δ*_C_ ranging 0 from to 190 ppm. The spectra were processed using JOEL v6.3.0 software, and the chemical shift (*δ*) values are expressed in ppm.

**LC–MS/MS analysis and Global Natural Product Social-aided dereplication of constituents from AL1.** In this study, high-resolution mass spectrometry was used to obtain tandem mass spectra, which were analyzed and clustered to create molecular networks and annotations of molecules in the database. The metabolomic mass profiles of AL1 collected from the Hokkaido region were screened using Global Natural Product Social (GNPS) based on MS/MS data in positive ionization mode. The nodes in the molecular network represent chemically related metabolites that are clustered together. The network was displayed as nodes reflecting the parent ion of each analyzed AL sample (Supplemental Information: Figure S1). LC-MS was used for the metabolite profiling of the LDAI-bioactive AL1 extract. Metabolite screening of the LC-MS/MS datasets of AL1 was performed using LC-MS Orbitrap, and the results are presented as 3D images obtained through DFF (Supplemental Information: Figures S2–S4). From the LC-MS/MS data analysis of bioactive AL1, we identified 12 ion parents with major compound carbohydrates and iridoid glucoside, as indicated by both the LC-MS/MS and 3D data (Figure 8, Table 4). Further analysis demonstrated the presence of minor constituent organic acids, amino acids, and organosulfur compounds identified using LC-MS/MS analysis and GNPS (Supplemental Information: Figure S1). GNPS, a global interactive online platform, was used as a powerful resource for understanding the molecular landscape of foods, including a mass spectrometry search tool (MASST) coupled with a reference database of food metabolites. Additionally, we performed LC–MS/MS analysis and GNPS-aided dereplication of several metabolites in selected AL extracts (**1**–**30**) including 18 constituents (**1**–**18**) from AL1. Metabolite dereplication revealed that 24 parent ions matched the known metabolites in the GNPS library, except for unidentified ion parents (**1**–**4**, **11**, and **12**). The GNPS molecular cluster generated from AL1 contains ion parents. Using LC-MSMS data and MN analyses, the unidentified ion parents in GNPS showed characteristic fragmentations. Cluster 1: ion parent (**2**) 424.4102 (MS1), 407.2867 (MS2), and 371.3444 (MS3); ion parent (**3**) 291.0977 (MS1), 291.0977 (MS2), and 144.9228 (MS3); ion parent (**4**) 360.1461 (MS1), 324.9067 (MS2), and 289.0067 (MS3); ion parent (**12**) 452.4411 (MS1), 435.3180 (MS2), and 399.4792 (MS3); Cluster 2: ion parent (**1**) 435.4147 (MS1), 359.3890 (MS2) and 148.9953, 134.9374 (MS3) and ion parent (**11**) 433.3988 (MS1), 375.3741 (MS2) and 357.3410 (MS3). The same ion parents, 360.1461 (**4**), 104.1058 (**7**), 356.3483 (**9**), and 391.2800 (**10**), were identified for the three bioactive extracts AL1, AL3, and AL6, respectively, while only ion parent 282.2759 (**13**) was identified for both AL3 and AL6. The following ion parents were unique for each extract, 435.4147: (**1**), 424.4102 (**2**), 291.0977 (**3**), 116.0693 (**5**), 258.1073 (**6**), 433.3988 (**11**) and 452.441 (**12**) for AL1, 338.3378 (**14**) for AL3, and ion parents 198.0951 (**15**), 180.0847 (**16**), 203.0504 (**17**), and 219.0242 (**18**) for AL6. (Supporting Information, Figure S1, and Table 4). Complementary 1D NMR measurements were performed for the bioactive extracts.

**Nuclear magnetic resonance 1D profiling and fingerprinting of AL1.** In this study, ^1^H-NMR profiles and ^13^C-NMR metabolite dereplication for AL1 were obtained. ^1^H-NMR spectra are shown in Figure 8A. The specific chemical shift between 6 and 9 ppm in the ^1^H-NMR spectrum of AL1 indicated the presence of carbohydrate moieties. Metabolite profiling using ^1^H-NMR and fingerprinting of AL1 revealed signatures of metabolites related to organosulfur compounds. NMR measurements of AL1 indicated that the chemical shifts corresponded to iridoid glucoside moiety derivative compounds (Figure 8A). The ^1^H-NMR profiles indicated the presence of a carbohydrate moiety, with a chemical shift between 2.5 and 6 ppm. Moreover, the ^1^H-NMR spectrum of AL1 showed characteristic chemical shifts, indicating peaks of an iridoid glucoside moiety. Previous reports have suggested that Allium species contain several components, including saponin derivatives, some of which have organosulfur compound derivatives as one of their major constituents [19]. The ^13^C-NMR data revealed the presence of carbohydrates and iridoid glucoside compounds in the extracts. Metabolome analysis of the AL1 methanolic extract allowed chemical composition screening using ^13^C-NMR and Dept (135 and 90) (Figure 8B). In this study, the MixONat method was utilized to analyze databases DB1–4, which contain ^13^C-NMR data for natural products extracted from the scientific literature [35,36]. The top 50 compounds (S**1**–S**50**) were matched with ^13^C-NMR chemical shifts; Dept (135 and 90) and intensities indicated the abundance of the metabolites. These 50 substances were then ranked based on their scores by comparing 1500 natural products from DB1–4 and their spectroscopic information. Dereplication revealed a variety of basic carbohydrates and glucoside-type molecules, including iridoid glucosides as well as minor amino acids, organosulfur compounds and organic acids such as the antioxidant ascorbic acid (KA2 **=** S**13**), and their derivatives S**5**−S**10**, suggesting that AL1 could be a potential resource for the development of functional foods and in drug discovery targeting MAFLD/MASH and other related diseases (Supplemental Information: Figures S5–S9).

Similar to the NMR results, HPLC chromatogram-specific peaks were observed for AL1 (Figures S10–S13). Subsequently, HPLC and LC-MS profiling of the selected bean samples was performed, and prominent peaks were observed for AL1 compared to AL2–9; thus, LC–MS/MS analysis using a diagnostic fragmentation filter (DFF) and Global Natural Product Social-aided dereplication of constituents of AL1 indicated unidentified ion parents (**1**–**4**, **11** and **12**). The MS fragmentation characteristics of unidentified ion parents **1**–**4**, **11**, and **12** suggested the detection of iridoid glucoside-type compounds in AL1. NMR, LC-LTQ-MS-MS, and MN analyses of the metabolome of the bioactive AL1 extract resulted in the tentative profiling and rapid identification of carbohydrates, iridoid glucoside, organic acids, amino acids, and organosulfur compounds using an NMR-based MixONat approach, suggesting this to be a chemical metabolite signature of the bioactive AL1 extract.

## 4. Conclusions

The current study explored the inhibitory effect of *Allium* extract on the accumulation of lipid droplets in FFA-loaded HepG2 cells using a strategy to identify antioxidants and LDAI/oxLDAI candidates from natural product extracts and food-derived compounds that have been previously developed. AL1, AL3, and AL6 were found to show a significant reduction in lipid droplet accumulation in hepatocytes. Furthermore, AL1, the most bioactive *Allium* extract, was found to inhibit TAG and TGOOH accumulation of in hepatocytes using a lipidomic approach. This inhibition was quantified under OA and LA conditions using LC-MS/MS analysis. The chemical constituents of AL1 contain mainly carbohydrates and glucosides, including iridoid glucosides, as well as minor organic acids, amino acids, and organosulfur metabolites, which could serve as promising targets for the discovery of LDA and oxLD regulator candidates. Limited studies exist on food-based functional components that effectively prevent MAFLD and metabolic dysfunction-associated steatohepatitis (MASH). Additional research is necessary for AL3 and AL6, which exhibited LDAI and antioxidant activity index values comparable to AL1. This study should concentrate on clarifying the structure–activity relationships to enhance our comprehension of the fundamental mechanisms of action of various compound types. Overall, the findings suggest that *Allium* may be an effective food for preventing excessive accumulation of lipid droplets and may serve as a potential source for drug discovery and development.

## Figures and Tables

**Table 1:** Antioxidants and LDAI/oxLDAI candidates (* μM).

Samples	Cell Viability		LDAI/oxLDAI		Antioxidant Activity	
	Cytotoxicity	Lipocytotoxicity	100 (μg/mL)	200 (μg/mL)	IC_50_ (μg/mL)	AAI
AL1	604.0	>800	44.0	92.5	108	1.9
AL2	404.2	652.3	0	0	181	1.1
AL3	675.2	745.6	0	93.5	54.0	3.7
AL4	475.4	835.6	0	10	23.1	8.6
AL5	814.1	895.5	0	0	198	1.0
AL6	721.4	981.7	0	81.0	75.4	2.6
AL7	939.1	940.1	0	0	103	1.9
AL8	683.3	892.5	0	80.0	200	1.0
AL9	>1000	>1000	0	0	500	0.4
KA1	>250	>500	0	40.2	20 *	10
KA2	>250	>500	0	0	10 *	20

**Table 2:** Comparison of accumulated TAG and TGOOH species under OA conditions and inhibited effect induced by AL1 ( ): 300 μg/mL.

OA	Neutral Lipid Species			
	TAG	TGOOH	TG(OOH)_2_	TG(OOH)_3_
Number accumulated	51	8	11	7
Number inhibited	9 (13)	1	0	2

**Table 3:** Comparison of accumulated TAG and TGOOH species under LA conditions and inhibited effect induced by AL1 ( ): 300 μg/mL.

LA	Neutral Lipid Species			
	TAG	TGOOH	TG(OOH)_2_	TG(OOH)_3_
Number accumulated	53	13	10	15
Number inhibited	0 (6)	6	1	6

**Table 4:** List of RT (min) and MSn (*m*/*z*) of AL1, AL3 and AL6 using LC-MS/MS analysis.

		AL1				AL3					AL6			
	RT (min)	MS1 (*m*/*z*)	MS2 (*m*/*z*)	MS3 (*m*/*z*)		RT (min)	MS1 (*m*/*z*)	MS2 (*m*/*z*)	MS3 (*m*/*z*)		RT (min)	MS1 (*m*/*z*)	MS2 (*m*/*z*)	MS3 (*m*/*z*)
**1**	0.01	435.4147	359.3890	148.9953 134.9374	**7**	2.25	104.1058	60.0242	could not detect	**4**	1.87	360.1464	325.0564	288.9400
**2**	0.01	424.4102	407.2867	371.3444	**8**	10.53	330.3329	312.3188	102.0371	**15**	1.94	198.0951	179.9797	162.9164
**3**	1.80	291.0977	161.9223	144.9228	**13**	10.93	282.2759	265.128	247.2555	**16**	1.94	180.0847	162.8774	145.0766
**4**	1.88	360.1461	324.9067	289.0067	**9**	10.93	356.3483	338.3411	101.8994	**17**	2.08	203.0504	185.0148	166.9430
**5**	1.93	116.0693	69.9151	could not detect	**10**	12.53	391.2800	148.8773	121.0314	**18**	2.14	219.0242	158.9557 176.7700	98.8628 (158.9557) 159.1721 (176.7700)
**6**	1.96	258.1073	103.9791	59.9807	**14**	12.9	338.3378	148.8773	121.0314	**7**	2.27	104.1058	59.9128	could not detect
**7**	2.26	104.1058	60.0242	could not detect						**8**	10.56	330.3330	312.2895	102.0410
**8**	10.41	330.3329	312.3188	102.0371						**9**	10.79	356.3484	338.3188	101.9674
**9**	10.90	356.3483	338.3411	101.8994						**13**	10.93	282.2759	265.218 4247.2157	247.2726 (265.2184) 163.1118 (247.2157)
**10**	12.51	391.2800	148.8773	121.0314						**10**	12.48	391.2801	148.9516	121.0154
**11**	12.96	433.3988	375.3741	357.3410										
**12**	13.12	452.4411	435.3180	399.4792										

## Data Availability

Data are contained within the article and Supplementary Files.

## Supplementary Materials

The following supporting information can be downloaded at: https://www.mdpi.com/article/10.3390/antiox13111310/s1: Materials and Methods: 1. Chemicals and instruments; 2. Liquid chromatography/mass spectrometry profiling of Allium extract; 3. Lipid droplet accumulation inhibition assay; 4. Metabolite fingerprinting via DFF and ^1^H-NMR analyses of selected Allium extracts; 5. Metabolite identification using DFF and ^1^H-NMR analyses of Allium extracts (ALs); Table S1: List of selected Allium extracts used in this study; Table S2: LC/MS data of detected triacylglycerol species (treated with OA); Table S3: LC/MS data of detected triacylglycerol species (treated with LA); Table S4: Detected hydroperoxide of triacylglycerol species (treated with OA); Table S5: Detected hydroperoxide of triacylglycerol species (treated with LA); Figure S1: Molecular networking of AL1-AL9; Figure S2: LC-MS profiling of bioactive AL1 extract. (A) Diagnostic Fragmentation Filtering (DFF) plot for metabolite analysis. (B) *m*/*z* list MS (n = 3) of AL1; (B) 3D visualization of MS data of AL1; Figure S3: LC-MS profiling of bioactive AL3 extract. (A) Diagnostic Fragmentation Filtering (DFF) plot for metabolite analysis. (B) *m*/*z* list MS (n = 3) of AL3; (B) 3D visualization of MS data of AL3; Figure S4: LC-MS profiling of bioactive AL6 extract. (A) Diagnostic Fragmentation Filtering (DFF) plot for metabolite analysis. (B) *m*/*z* list MS (n = 3) of AL6; (B) 3D visualization of MS data of AL6; Figure S5: Dereplication analysis from MixONat, structure of top 50 metabolites: compounds S**1**–S**10**; Figure S6: Dereplication analysis from MixONat, structure of top 50 metabolites: compounds S**16**–S**25**; Figure S7: Dereplication analysis from MixONat, structure of top 50 metabolites: compounds S**26**–S**35**; Figure S8: Dereplication analysis from MixONat, structure of top 50 metabolites: compounds S**36**–S**40** and S**11**–S**15**; Figure S9: Dereplication analysis from MixONat, structure of top 50 metabolites: compounds S**41**–S**50**; Figure S10: HPLC spectra of AL1 and AL6 (Left: AL1, Right: AL6); Figure S11: ^1^H NMR spectrum of AL1 in DMSO; Figure S12: ^1^H NMR spectrum of AL1 and AL6 in DMSO; Figure S13: ^1^H-NMR spectra: (a) spectra of AL3 and (b) spectra of AL6. (c) Comparison of ^1^H-NMR spectra of AL3 and AL6; Figure S14: Mixture analysis LC-MS/MS experimental flow. Picture S1. List and pictures of parts of Allium AL1–9 used in the study. Video S1: Real-time LD Accumulation and Inhibition by AL1.

## Abbreviations

LDA, lipid droplet accumulation; LDAI, lipid droplet accumulation inhibition; MAFLD, metabolic dysfunction-associated fatty liver disease; MASH, metabolic dysfunction-associated steatohepatitis; oxLD, oxidized lipid droplet; OA, oleic acid; LA, linoleic acid; LD, lipid droplet; TAG, triacylglycerol; TGOOH, triacylglycerol hydroperoxide; FFA, free fatty acid; LC/MS, liquid chromatography/mass spectrometry; NMR, nuclear magnetic resonance; HPLC, high-performance liquid chromatography; DFF, diagnostic fragmentation filter.
