# Supplementary material for: Inhibition of Accumulation of Neutral Lipids and Their Hydroperoxide Species in Hepatocytes by Bioactive Allium sativum Extract
Source: Antioxidants (Basel). 2024 Oct 28;13(11):1310. doi: 10.3390/antiox13111310 (PMC11591070; doi:10.3390/antiox13111310)
Supplement: Supplementary file 1 [file antioxidants-13-01310-s001.zip › Supplementary Information-2024-[Dibwe DF-Allium AL1] proofreading/Antioxidant-Supplementary Information-2024-[Dibwe DF-Allium AL1]-Prof Hui-Dibwe For Submission 20240921-proofreading 20241027.pdf]

Supporting Information

Inhibition of neutral lipid accumulation and their hydroperoxide species in hepatocytes by bioactive *Allium sativum* extract

Dya Fita Dibwe [1], Saki Oba [2], Satomi Monde [2], and Shu-Ping Hui [1,*]

[1] Faculty of Health Sciences, Hokkaido University, Kita-12, Nishi-5, Kita-Ku, Sapporo 060-0812, Japan;
[2] Graduate School of Health Sciences, Hokkaido University, Kita-12, Nishi-5, Kita-Ku, Sapporo 060-0812, Japan;

* Correspondences: keino@hs.hokudai.ac.jp; Tel./Fax: +81-11-706-369

Supporting Information

Table of contents:

Materials and Methods P4

1. Chemicals and instruments P4

2. Liquid chromatography/mass spectrometry profiling of *Allium* extract P5

3. Lipid droplet accumulation inhibition assay. P5

4. Metabolite fingerprinting via DFF and, ^{1}H-NMR analyses of selected *Allium* P6

5. Metabolite profiling via DFF and, ^{1}H-NMR analyses of selected *Allium* extracts P7

Table S1. List of selected *Allium* used in this study. P8

Table S2. LC/MS data of detected triacylglycerol species (treated with OA). P9

Table S3. LC/MS data of detected triacylglycerol species (treated with LA). P10

Table S4. Detected hydroperoxide of triacylglycerol species (treated with OA). P11

Table S5. Detected hydroperoxide of triacylglycerol species (treated with LA). P13

Figure S1. Molecular networking of AL1–AL9 P15

Figure S2. LC-MS profiling of bioactive AL1 extract. (A) Diagnostic Fragmentation Filtering (DFF) plot for metabolites analysis. (B) *m/z* list MS(n=3) of AL-1 (B) 3D visualization of MS data of AL1. P16

Figure S3. LC-MS profiling of bioactive AL3 extract. (A) Diagnostic Fragmentation Filtering (DFF) plot for metabolites analysis. (B) *m/z* list MS(n=3) of AL3 (B) 3D visualization of MS data of AL3. P17

Figure S4. LC-MS profiling of bioactive AL6 extract. (A) Diagnostic Fragmentation Filtering (DFF) plot for metabolites analysis. (B) *m/z* list MS(n=3) of AL6 (B) 3D visualization of MS data of AL6. P18

Figure S5. Dereplication analysis from MixONat, structure of top 50 metabolites: compounds S**1**–S**10**. P19

Figure S6. Dereplication analysis from MixONat, structure of top 50 metabolites: compounds S**16**–S**25**. P20

Figure S7. Dereplication analysis from MixONat, structure of top 50 metabolites: compounds S**26**–S**35.** P21

Figure S8. Dereplication analysis from MixONat, structure of top 50 metabolites: compounds S**31**–S**40** P22

Figure S9. Dereplication analysis from MixONat, structure of top 50 metabolites: compounds S**41**–S**50.** P23

Figure S10. HPLC spectra of AL1 and AL6 (Left: AL1, Right: AL6). P24

Figure S11. ^{1}H NMR spectrum of AL1 in DMSO. P25

Figure S12. NMR profile of AL1 and AL6. P26

Figure S13. ^{1}H-NMR spectra (a) Spectra group AL3 and (b) Spectra group AL6 (c) Comparison of ^{1}H-NMR spectra of the AL3 and AL6. P27

Figure S14. Mixture analysis LC-MS/MS experimental flow. P28

Materials and Methods

1. *Chemicals and instruments*

General Experimental Procedures: NMR spectroscopy was conducted on a JEOL ECX400 Delta instrument using TMS as the internal reference; chemical shifts were reported as δ values. HR-ESI-MS analysis was performed using an LTQ Orbitrap XL device (Thermo Fisher Scientific Inc., San Jose, CA, USA). Methanol was obtained from Wako. Cell culture materials, including high-glucose DMEM, DPBS, trypsin EDTA, FBS, and penicillin-streptomycin (100 U/mL), were sourced from Gibco (Life Technologies, Carlsbad, CA, USA). Additional cell culture supplies were procured from Corning (NY, USA). NMR data were collected using a 400 MHz JNM-ECX400P instrument (JOEL, Japan). Spectral processing was performed using the JOEL software, and chemical shifts (δ) were expressed in ppm. OA was acquired from Cayman Chemical (Ann Arbor, MI, USA), and the absorbance was measured using ARVO-MX (Perkin Elmer, Waltham, MA, USA) following previously described methods (22, 25,26)

2. *Liquid chromatography/mass spectrometry profiling of Allium sativum extract*

LC-MS instrument conditions

An Atlantis T3 C18 column (2.1×150 mm, 3 μm, 155 Waters, Milford, MA, USA) was used to separate the ALs samples at a flow rate of 200 μL/min. LC gradient elution employed a mobile phase composed of 10 mM ammonium acetate solution, isopropanol, and methanol. Measurements were conducted in the positive mode, with the MS capillary voltage set at 4.04 kV. The sheath gas (nitrogen) flow rate was 50 psi, whereas the auxiliary gas (nitrogen) was maintained at 20 psi. High-resolution MS data were acquired within the scan range of m/z 150–1100. Data-dependent acquisition using collision-induced dissociation (CID) in ion-trap mode was employed to obtain MS/MS spectra for low-resolution masses. As previously described (22, 25,26), Xcalibur 2.2 (Thermo Fisher Scientific Inc., San Jose, CA, USA) was used to process the raw data.

3. *Lipid droplet accumulation inhibition assay*

LDAI activity was determined using an Oil Red O assay with 24-well plates (n = 4 per treatment) based on the staining of LDs in cultured hepatocytes, according to the manufacturer's instructions. HepG2 cells (1.5×104/well) were supplemented with 10% FBS, cultured, seeded into 35 mm dishes, and treated with the tested samples after 24 h. Oil Red O, a fat-soluble dye, is widely used to stain neutral lipids in LDs, as previously described (22, 25,26). Next, quantification of LD inhibition was assessed for test BEs by comparing it to the untreated control group (+OA) and normalizing the LDA absorbance values (%), as previously reported (22, 25,26). LD staining assay. Staining was performed as previously described, with modifications (22, 25,26).

4. *Real-time Lipid droplet accumulation inhibition assay*

Real time LDA inhibition of AL1: 24 hours after cell seeding, OA and the sample were added and real-time images were recorded. Twelve hours after OA addition, lipid droplet accumulation was observed in the control group. In contrast, in the treated cells, lipid droplet accumulation was suppressed after 12 h of treatment. Double staining of hepatocytes. Oil Red O staining was used to stain the LDs, and Hoechst staining was used to stain nuclei. The red color represents lipid droplets, the blue color represents the cell nucleus, and the lower part is a merged image. Fluorescence staining assay. The staining was done with modifications based on Tsukui et al. [27] HepG2 cells were seeded in 35 mm glass-bottomed dishes at 4.0×10^5/dish. After 24 hours of incubation, OA (0.25 mM) and samples (150 µg/mL and 300 µg/mL) were treated. 24 h after treatment, Oil Red O staining was performed as in previously reported (22,25,26), and then a solution of Hoechst's reagent mixed with PBS (Hoechst: PBS = 1:1000) was added. Specifically, after Oil Red O staining using the same procedure previously reported (22,25,26) instead of adding 100% IPA, a mixed solution of Hoechst-PBS was added and incubated in the dark for 25 minutes. The images were then captured using a BZ-9000 fluorescence microscope (Keyence Co., Ltd., Osaka, Japan). The filters used were TRITC and DAPI-B (both from Keyence Co., Ltd., Osaka, Japan).

5. *Metabolite profiling via DFF and, ^{1}H-NMR analyses of selected Allium extracts*

Typically, natural products are produced as a combination of structurally related compounds rather than individual substances. Due to their shared structural features, many compounds within the same class undergo similar MS/MS fragmentation, resulting in several identical product ions and/or neutral losses. DFF aims to precisely identify all compounds of a particular class in a complex extract by screening non-targeted LC-MS/MS datasets for MS/MS spectra containing class-specific product ions and/or neutral losses (22, 25,26). The 3D LC-MS plot, which displays the retention time and MS values, illustrates the LC-MS profile of the methanolic extract. Analysis of AL1 using LC-MS primarily revealed carbohydrate and glucoside metabolites, including iridoid glucosides, along with minor organic acids, amino acids, and organosulfur metabolites.

The primary chemical components of the extract were identified as major carbohydrates and iridoid glucosides, along with minor organic acids, amino acids, and organosulfur compounds, when compared with in-house standards. Differential fragmentation filtering (DFF) offers a quick and efficient method for detecting entire classes of compounds in natural food product mixtures. This technique is particularly useful for natural product dereplication and discovery. DFF was employed to analyze metabolites from the LC-MS/MS datasets of AL1. The results were presented as 3D images and their corresponding DFF. The 3D LC-MS comparison of AL1, AL3 and AL6 using the DFF approach, as illustrated in the graph displaying the characteristic ion products and precursors from LC-MS/MS analysis.

All statistical analyses were performed as follows: GraphPad Prism V7.0/10.1.2. One-way ANOVA with Tukey's multiple comparison test was used for multiple comparisons. A significance level of 5 % %was used. Results are presented as the mean ± standard deviation (SD).

Table S1. List of selected *Allium* used in this study.

No	Species	abbr.	Family	Local name	Consumed/used as	The part used in this study
**1**	*Allium sativum*	AL1	*Allium*	Ninniku	cooked, raw	bulb
**2**	*Allium victorialis*	AL2	*Allium*	Gyoujaninnniku	cooked, raw	leaves
**3**	*Allium victorialis*	AL3	*Allium*	Gyoujaninnniku	cooked, raw	stem
**4**	*Allium schoenoprasum var. foliosum*	AL4	*Allium*	Asatuki	cooked, raw	leaves
**5**	*Allium schoenoprasum var. foliosum*	AL5	*Allium*	Asatuki	cooked, raw	stem
**6**	*Allium cepa*	AL6	*Allium*	Kitamanegi	cooked, raw	bulb
**7**	*Allium cepa*	AL7	*Allium*	Sinntamanegi	cooked, raw	bulb
**8**	*Allium cepa*	AL8	*Allium*	Sirotamanegi	cooked, raw	bulb
**9**	*Allium cepa*	AL9	*Allium*	Akatamanegi	cooked, raw	bulb

Table S2. LC/MS data of detected triacylglycerol species (treated with OA).

Lipid species	RT	Ion	calc. *m/z*	exptl. *m/z*	ppm	Lipid species	RT	Ion	calc. *m/z*	exptl. *m/z*	ppm
TG42:0	13.42	$[M+NH_4]^+$	740.6763	740.6774	1.49	TG54:0	15.82	$[M+NH_4]^+$	908.8641	908.8655	1.54
TG44:0	13.85	$[M+NH_4]^+$	768.7076	768.7087	1.43	TG54:1	15.39	$[M+NH_4]^+$	906.8484	906.8477	–0.77
TG46:0	14.20	$[M+NH_4]^+$	796.7389	796.7398	1.13	TG54:2	15.10	$[M+NH_4]^+$	904.8328	904.8336	0.88
TG46:1	13.90	$[M+NH_4]^+$	794.7232	794.7244	1.51	TG54:3	14.90	$[M+NH_4]^+$	902.8171	902.8178	0.78
TG46:2	13.60	$[M+NH_4]^+$	792.7076	792.7081	0.63	TG54:4	14.53	$[M+NH_4]^+$	900.8015	900.8016	0.11
TG46:3	13.31	$[M+NH_4]^+$	790.6919	790.6923	0.51	TG54:5	14.26	$[M+NH_4]^+$	898.7858	898.7858	0.00
TG48:0	14.58	$[M+NH_4]^+$	824.7702	824.7717	1.82	TG54:6	14.13	$[M+NH_4]^+$	896.7702	896.7698	–0.45
TG48:1	14.26	$[M+NH_4]^+$	822.7545	822.7558	1.58	TG54:7	13.94	$[M+NH_4]^+$	894.7545	894.7548	0.34
TG48:2	13.99	$[M+NH_4]^+$	820.7389	820.7396	0.85	TG54:8	13.63	$[M+NH_4]^+$	892.7389	892.7395	0.67
TG48:3	13.70	$[M+NH_4]^+$	818.7232	818.7234	0.24	TG56:4	14.93	$[M+NH_4]^+$	928.8328	928.8315	–1.40
TG48:5	11.27	$[M+NH_4]^+$	814.6169	814.6190	2.58	TG56:5	14.58	$[M+NH_4]^+$	926.8171	926.8160	–1.19
TG50:0	14.99	$[M+NH_4]^+$	852.8015	852.8041	3.05	TG56:6	14.47	$[M+NH_4]^+$	924.8015	924.8008	–0.76
TG50:1	14.60	$[M+NH_4]^+$	850.7858	850.7871	1.53	TG56:7	14.24	$[M+NH_4]^+$	922.7858	922.7859	0.11
TG50:2	14.33	$[M+NH_4]^+$	848.7702	848.7712	1.18	TG56:8	14.03	$[M+NH_4]^+$	920.7702	920.7707	0.54
TG50:3	14.10	$[M+NH_4]^+$	846.7545	846.7550	0.59	TG58:10	13.89	$[M+NH_4]^+$	944.7702	944.7710	0.85
TG50:4	13.83	$[M+NH_4]^+$	844.7389	844.7390	0.12	TG58:11	13.60	$[M+NH_4]^+$	942.7545	942.7537	–0.85
TG50:5	13.65	$[M+NH_4]^+$	842.7232	842.7231	–0.12	TG58:6	14.90	$[M+NH_4]^+$	952.8328	952.8326	–0.21
TG50:6	13.45	$[M+NH_4]^+$	840.7076	840.7075	–0.12	TG58:7	14.51	$[M+NH_4]^+$	950.8171	950.8157	–1.47
TG52:0	15.39	$[M+NH_4]^+$	880.8328	880.8357	3.29	TG58:8	14.36	$[M+NH_4]^+$	948.8015	948.8016	0.11
TG52:1	15.02	$[M+NH_4]^+$	878.8171	878.8176	0.57	TG58:9	14.13	$[M+NH_4]^+$	946.7858	946.7860	0.21
TG52:2	14.63	$[M+NH_4]^+$	876.8015	876.8026	1.25	TG60:10	14.20	$[M+NH_4]^+$	972.8015	972.8021	0.62
TG52:3	14.44	$[M+NH_4]^+$	874.7858	874.7862	0.46	TG60:11	14.06	$[M+NH_4]^+$	970.7858	970.7865	0.72
TG52:4	14.17	$[M+NH_4]^+$	872.7702	872.7702	0.00	TG60:12	13.83	$[M+NH_4]^+$	968.7702	968.7744	4.34
TG52:5	14.03	$[M+NH_4]^+$	870.7545	870.7544	–0.11	TG62:12	14.12	$[M+NH_4]^+$	996.8015	996.8027	1.20
TG52:6	13.81	$[M+NH_4]^+$	868.7389	868.7390	0.12	TG62:13	13.94	$[M+NH_4]^+$	994.7858	994.7869	1.11
TG52:7	13.54	$[M+NH_4]^+$	866.7232	866.7240	0.92						

Table S3. LC/MS data of detected triacylglycerol species (treated with LA).

Lipid species	RT	Ion	calc. *m*/*z*	exptl. *m*/*z*	ppm	Lipid species	RT	Ion	calc. *m*/*z*	exptl. *m*/*z*	ppm
TG42:0	13.46	$[M+NH_4]^+$	740.6763	740.6771	1.08	TG54:1	15.46	$[M+NH_4]^+$	906.8484	906.8491	0.77
TG44:0	13.84	$[M+NH_4]^+$	768.7076	768.7084	1.04	TG54:2	15.17	$[M+NH_4]^+$	904.8328	904.8333	0.55
TG46:0	14.34	$[M+NH_4]^+$	796.7389	796.7397	1.00	TG54:3	14.90	$[M+NH_4]^+$	902.8171	902.8173	0.22
TG46:1	13.91	$[M+NH_4]^+$	794.7232	794.7240	1.01	TG54:4	14.63	$[M+NH_4]^+$	900.8015	900.8019	0.44
TG46:2	13.64	$[M+NH_4]^+$	792.7076	792.7084	1.01	TG54:5	14.34	$[M+NH_4]^+$	898.7858	898.7865	0.78
TG46:3	13.33	$[M+NH_4]^+$	790.6919	790.6922	0.38	TG54:6	14.02	$[M+NH_4]^+$	896.7702	896.7710	0.89
TG48:0	14.70	$[M+NH_4]^+$	824.7702	824.7715	1.58	TG54:7	13.75	$[M+NH_4]^+$	894.7545	894.7553	0.89
TG48:1	14.40	$[M+NH_4]^+$	822.7545	822.7558	1.58	TG54:8	13.53	$[M+NH_4]^+$	892.7389	892.7394	0.56
TG48:2	14.09	$[M+NH_4]^+$	820.7389	820.7398	1.10	TG56:10	13.46	$[M+NH_4]^+$	916.7389	916.7396	0.76
TG48:3	13.72	$[M+NH_4]^+$	818.7232	818.7242	1.22	TG56:4	14.99	$[M+NH_4]^+$	928.8328	928.8325	◎0.32
TG48:4	13.44	$[M+NH_4]^+$	816.7076	816.7078	0.24	TG56:5	14.66	$[M+NH_4]^+$	926.8171	926.8163	◎0.86
TG50:0	15.06	$[M+NH_4]^+$	852.8015	852.8031	1.88	TG56:6	14.40	$[M+NH_4]^+$	924.8015	924.8008	◎0.76
TG50:1	14.74	$[M+NH_4]^+$	850.7858	850.7874	1.88	TG56:7	14.16	$[M+NH_4]^+$	922.7858	922.7846	◎1.30
TG50:2	14.45	$[M+NH_4]^+$	848.7702	848.7719	2.00	TG56:8	13.89	$[M+NH_4]^+$	920.7702	920.7698	◎0.43
TG50:3	14.16	$[M+NH_4]^+$	846.7545	846.7562	2.01	TG56:9	13.68	$[M+NH_4]^+$	918.7545	918.7543	◎0.22
TG50:4	13.82	$[M+NH_4]^+$	844.7389	844.7404	1.78	TG58:10	13.84	$[M+NH_4]^+$	944.7702	944.7706	0.42
TG50:5	13.57	$[M+NH_4]^+$	842.7232	842.7241	1.07	TG58:11	13.63	$[M+NH_4]^+$	942.7545	942.7546	0.11
TG50:6	13.31	$[M+NH_4]^+$	840.7076	840.7082	0.71	TG58:6	14.81	$[M+NH_4]^+$	952.8328	952.8318	◎1.05
TG52:0	15.46	$[M+NH_4]^+$	880.8328	880.8342	1.59	TG58:7	14.56	$[M+NH_4]^+$	950.8171	950.8163	◎0.84
TG52:1	15.12	$[M+NH_4]^+$	878.8171	878.8181	1.14	TG58:8	14.40	$[M+NH_4]^+$	948.8015	948.8013	◎0.21
TG52:2	14.81	$[M+NH_4]^+$	876.8015	876.8026	1.25	TG58:9	14.16	$[M+NH_4]^+$	946.7858	946.7856	◎0.21
TG52:3	14.56	$[M+NH_4]^+$	874.7858	874.7872	1.60	TG60:10	14.16	$[M+NH_4]^+$	972.8015	972.8019	0.41
TG52:4	14.23	$[M+NH_4]^+$	872.7702	872.7722	2.29	TG60:11	13.91	$[M+NH_4]^+$	970.7858	970.7863	0.52
TG52:5	13.86	$[M+NH_4]^+$	870.7545	870.7554	1.03	TG60:12	13.75	$[M+NH_4]^+$	968.7702	968.7708	0.62
TG52:6	13.63	$[M+NH_4]^+$	868.7389	868.7396	0.81	TG60:13	13.53	$[M+NH_4]^+$	966.7545	966.7554	0.93
TG52:7	13.46	$[M+NH_4]^+$	866.7232	866.7241	1.04	TG62:12	14.20	$[M+NH_4]^+$	996.8015	996.8010	◎0.50
TG52:8	13.08	$[M+NH_4]^+$	864.7076	864.7084	0.93	TG62:13	13.84	$[M+NH_4]^+$	994.7858	994.7875	1.71
TG54:0	15.83	$[M+NH_4]^+$	908.8641	908.8653	1.32	TG62:14	13.70	$[M+NH_4]^+$	992.7702	992.7709	0.71

Table S4. Detected hydroperoxide of triacylglycerol species (treated with OA).

**Lipid species**	**RT**	**Ion**	**calc. *m/z***	**exptl. *m/z***	**ppm**
TGOOH52:7	1.98	$[M+NH_4]^+$	898.7130	898.7126	◎0.45
TGOOH54:7	2.05	$[M+NH_4]^+$	926.7443	926.7438	◎0.54
TGOOH56:10	10.21	$[M+NH_4]^+$	948.7287	948.7275	◎1.26
TGOOH58:10	10.86	$[M+NH_4]^+$	976.7600	976.7565	◎3.58
TGOOH58:11	10.23	$[M+NH_4]^+$	974.7443	974.7411	◎3.28
TGOOH60:14	10.82	$[M+NH_4]^+$	996.7287	996.7333	4.62
TGOOH60:15	10.21	$[M+NH_4]^+$	994.7130	994.7175	4.52
TGOOH66:18	13.18	$[M+NH_4]^+$	1072.7600	1072.7618	1.68

**Lipid species**	**RT**	**Ion**	**calc. *m/z***	**exptl. *m/z***	**ppm**
$TG(-OOH)_2$ 46:2	14.15	$[M+NH_4]^+$	856.6872	856.6878	0.70
$TG(-OOH)_2$ 46:3	13.81	$[M+NH_4]^+$	854.6716	854.6718	0.23
$TG(-OOH)_2$ 48:2	14.54	$[M+NH_4]^+$	884.7185	884.7195	1.13
$TG(-OOH)_2$ 48:3	14.17	$[M+NH_4]^+$	882.7029	882.7031	0.23
$TG(-OOH)_2$ 48:4	14.03	$[M+NH_4]^+$	880.6872	880.6877	0.57
$TG(-OOH)_2$ 48:5	13.78	$[M+NH_4]^+$	878.6716	878.6724	0.91
$TG(-OOH)_2$ 50:2	14.97	$[M+NH_4]^+$	912.7498	912.7491	◎0.77
$TG(-OOH)_2$ 50:5	14.15	$[M+NH_4]^+$	906.7029	906.7034	0.55
$TG(-OOH)_2$ 50:6	13.87	$[M+NH_4]^+$	904.6872	904.6877	0.55
$TG(-OOH)_2$ 52:2	15.29	$[M+NH_4]^+$	940.7811	940.7808	◎0.32
$TG(-OOH)_2$ 52:3	14.97	$[M+NH_4]^+$	938.7655	938.7665	1.07
$TG(-OOH)_2$ 52:6	14.22	$[M+NH_4]^+$	932.7185	932.7189	0.43
$TG(-OOH)_2$ 52:7	14.12	$[M+NH_4]^+$	930.7029	930.7034	0.54
$TG(-OOH)_2$ 52:8	13.81	$[M+NH_4]^+$	928.6872	928.6873	0.11
$TG(-OOH)_2$ 54:2	15.66	$[M+NH_4]^+$	968.8124	968.8128	0.41
$TG(-OOH)_2$ 54:3	15.33	$[M+NH_4]^+$	966.7968	966.7961	◎0.72
$TG(-OOH)_2$ 54:8	14.15	$[M+NH_4]^+$	956.7185	956.7189	0.42

Lipid species	RT	Ion	calc. *m/z*	exptl. *m/z*	ppm
TG(-OOH)$_3$ 54:10	10.18	$[M+NH_4]^+$	984.6771	984.6758	◎1.32
TG(-OOH)$_3$ 54:3	13.70	$[M+NH_4]^+$	998.7866	998.7878	1.20
TG(-OOH)$_3$ 54:4	13.43	$[M+NH_4]^+$	996.7710	996.7726	1.61
TG(-OOH)$_3$ 56:10	10.81	$[M+NH_4]^+$	1012.7084	1012.7070	◎1.38
TG(-OOH)$_3$ 56:11	10.19	$[M+NH_4]^+$	1010.6927	1010.6912	◎1.48
TG(-OOH)$_3$ 58:11	10.88	$[M+NH_4]^+$	1038.7240	1038.7227	◎1.25
TG(-OOH)$_3$ 58:7	13.42	$[M+NH_4]^+$	1046.7866	1046.7837	◎2.77

Table S5. Detected hydroperoxide of triacylglycerol species (treated with LA).

**Lipid species**	**RT**	**Ion**	**calc. *m/z***	**exptl. *m/z***	**ppm**
TGOOH48:5	11.40	$[M+NH_4]^+$	846.6817	846.6847	3.54
TGOOH52:4	11.84	$[M+NH_4]^+$	904.7600	904.7596	◎0.44
TGOOH54:5	11.41	$[M+NH_4]^+$	930.7756	930.7767	1.18
TGOOH54:6	11.48	$[M+NH_4]^+$	928.7600	928.7604	0.43
TGOOH54:9	6.82	$[M+NH_4]^+$	922.7130	922.7124	◎0.65
TGOOH56:10	10.17	$[M+NH_4]^+$	948.7287	948.7265	◎2.32
TGOOH56:7	13.93	$[M+NH_4]^+$	954.7756	954.7752	◎0.42
TGOOH58:10	10.84	$[M+NH_4]^+$	976.7600	976.7582	◎1.84
TGOOH58:11	10.24	$[M+NH_4]^+$	974.7443	974.7421	◎2.26
TGOOH60:13	10.82	$[M+NH_4]^+$	998.7443	998.7407	◎3.60
TGOOH60:14	10.85	$[M+NH_4]^+$	996.7287	996.7334	4.72
TGOOH60:15	10.21	$[M+NH_4]^+$	994.7130	994.7176	4.62
TGOOH66:18	13.22	$[M+NH_4]^+$	1072.7600	1072.7616	1.49

**Lipid species**	**RT**	**Ion**	**calc. *m/z***	**exptl. *m/z***	**ppm**
TG(-OOH)$_2$ 48:5	13.67	$[M+NH_4]^+$	878.6716	878.6732	1.82
TG(-OOH)$_2$ 50:2	14.99	$[M+NH_4]^+$	912.7498	912.7510	1.31
TG(-OOH)$_2$ 50:6	13.80	$[M+NH_4]^+$	904.6872	904.6870	◎0.22
TG(-OOH)$_2$ 52:2	15.30	$[M+NH_4]^+$	940.7811	940.7806	◎0.53
TG(-OOH)$_2$ 52:3	15.06	$[M+NH_4]^+$	938.7655	938.7649	◎0.64
TG(-OOH)$_2$ 54:2	15.78	$[M+NH_4]^+$	968.8124	968.8132	0.83
TG(-OOH)$_2$ 54:3	15.35	$[M+NH_4]^+$	966.7968	966.7972	0.41
TG(-OOH)$_2$ 56:6	14.99	$[M+NH_4]^+$	988.7811	988.7799	◎1.21
TG(-OOH)$_2$ 58:7	15.02	$[M+NH_4]^+$	1014.7968	1014.7980	1.18
TG(-OOH)$_2$ 62:13	6.95	$[M+NH_4]^+$	1058.7655	1058.7658	0.28

**Lipid species**	**RT**	**Ion**	**calc. *m/z***	**exptl. *m/z***	**ppm**
TG(-OOH)$_3$ 50:3	12.59	$[M+NH_4]^+$	942.7240	942.7233	◎0.74
TG(-OOH)$_3$ 52:3	13.07	$[M+NH_4]^+$	970.7553	970.7550	◎0.31
TG(-OOH)$_3$ 52:4	12.69	$[M+NH_4]^+$	968.7397	968.7404	0.72
TG(-OOH)$_3$ 52:5	12.48	$[M+NH_4]^+$	966.7240	966.7243	0.31
TG(-OOH)$_3$ 54:10	10.22	$[M+NH_4]^+$	984.6771	984.6765	◎0.61
TG(-OOH)$_3$ 54:3	13.77	$[M+NH_4]^+$	998.7866	998.7881	1.50
TG(-OOH)$_3$ 54:4	13.52	$[M+NH_4]^+$	996.7710	996.7707	◎0.30
TG(-OOH)$_3$ 54:5	12.71	$[M+NH_4]^+$	994.7553	994.7553	0.00
TG(-OOH)$_3$ 54:6	12.41	$[M+NH_4]^+$	992.7397	992.7394	◎0.30
TG(-OOH)$_3$ 56:10	10.85	$[M+NH_4]^+$	1012.7084	1012.7072	◎1.18
TG(-OOH)$_3$ 56:11	10.22	$[M+NH_4]^+$	1010.6927	1010.6913	◎1.39
TG(-OOH)$_3$ 58:11	10.84	$[M+NH_4]^+$	1038.7240	1038.7235	◎0.48
TG(-OOH)$_3$ 58:7	13.44	$[M+NH_4]^+$	1046.7866	1046.7822	◎4.20
TG(-OOH)$_3$ 60:11	13.42	$[M+NH_4]^+$	1066.7553	1066.7515	◎3.56
TG(-OOH)$_3$ 66:18	12.25	$[M+NH_4]^+$	1136.7397	1136.7347	◎4.40

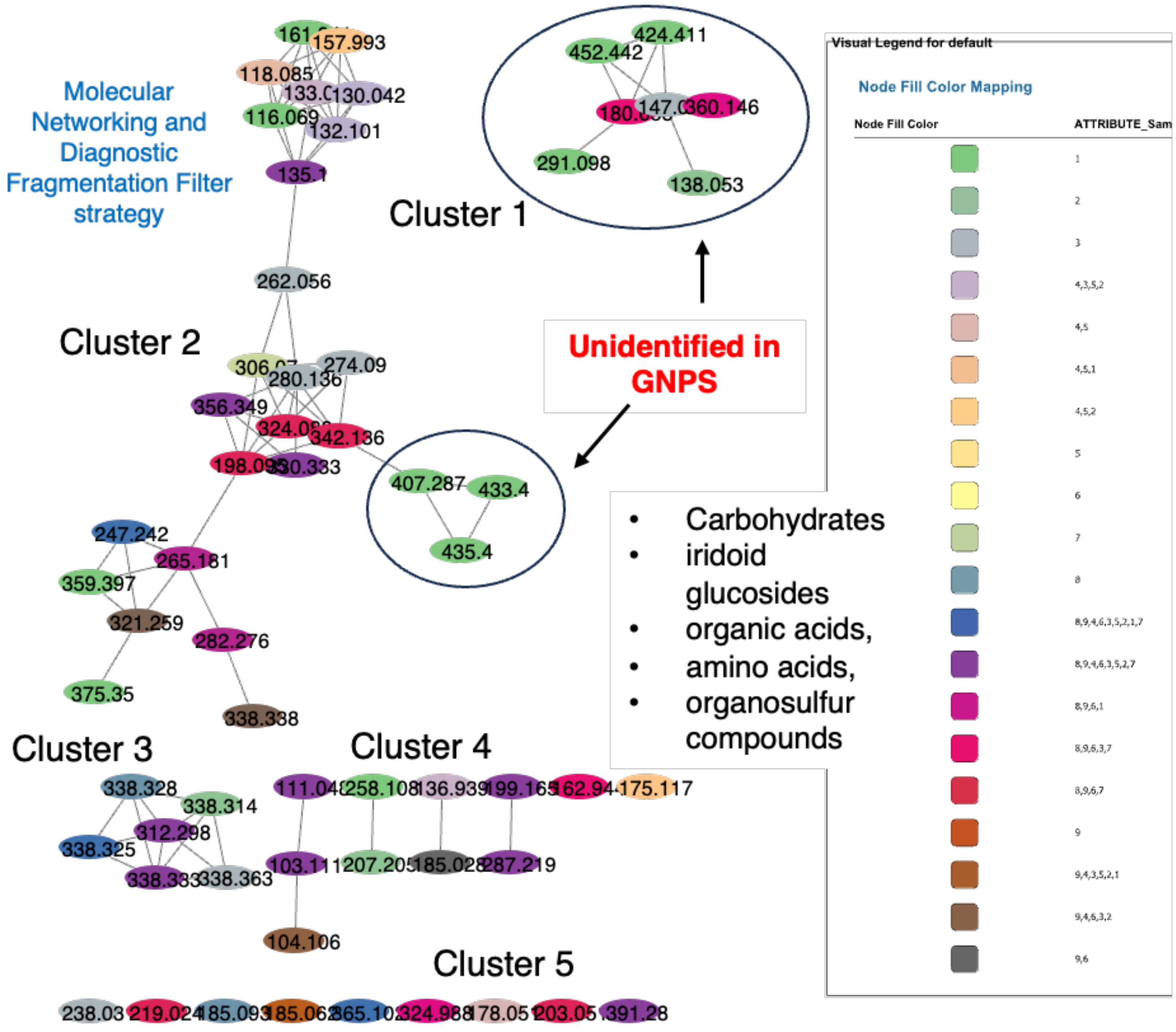

Figure S1. Molecular networking of AL1–AL9.

A

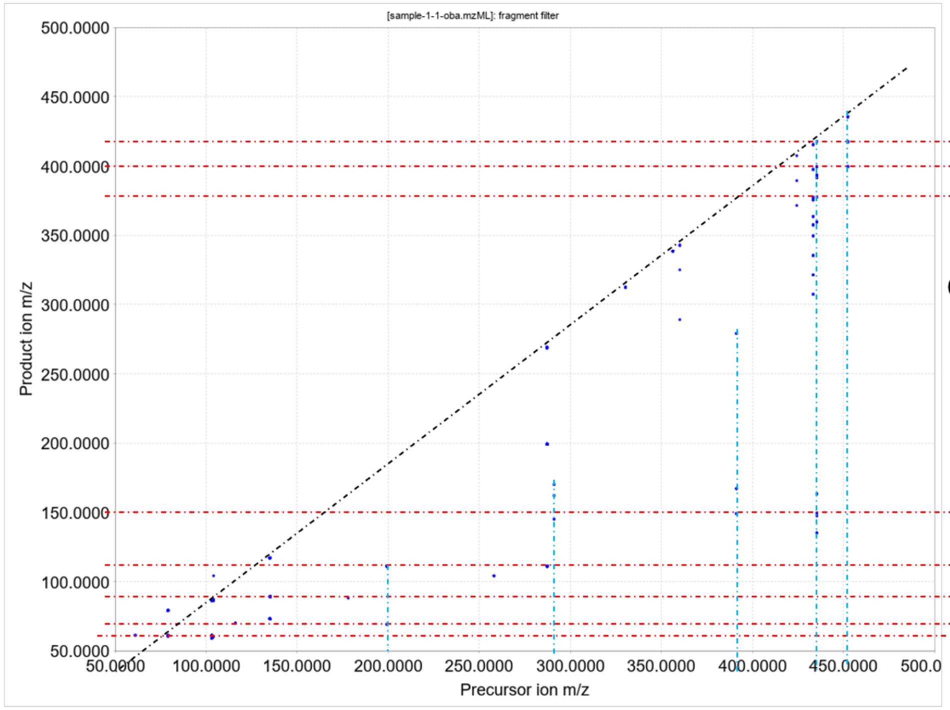

Represent the diagnostic product ions that are required to be considered a putative metabolites x
Represent the [M + H]+ precursor ions.

B

	Allium 1			
	RT (min)	MS1 (m/z)	MS2 (m/z)	MS3 (m/z)
1	0.01	435.4147	359.3890	148.9953<br>134.9374
2	0.01	424.4102	407.2867	371.3444
3	1.80	291.0977	161.9223	144.9228
4	1.88	360.1461	324.9067	289.0067
5	1.93	116.0693	69.9151	can't detect
6	1.96	258.1073	103.9791	59.9807
7	2.26	104.1058	60.0242	can't detect
8	10.41	330.3329	312.3188	102.0371
9	10.90	356.3483	338.3411	101.8994
10	12.51	391.2800	148.8773	121.0314
11	12.96	433.3988	375.3741	357.3410
12	13.12	452.4411	435.3180	399.4792

C

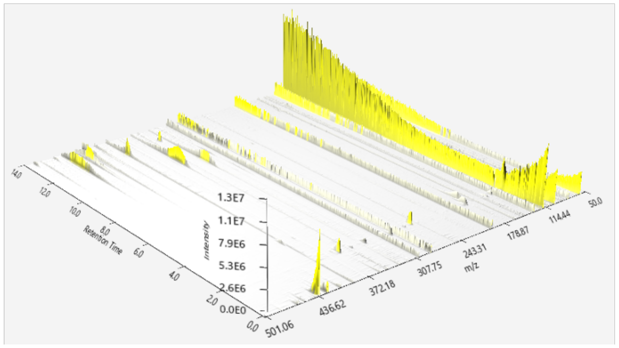

Figure S2. LC-MS profiling of bioactive AL1 extract. (A) Diagnostic Fragmentation Filtering (DFF) plot for metabolites analysis. (B) *m/z* list MS(n=3) of AL1 (B) 3D visualization of MS data of AL1.

A

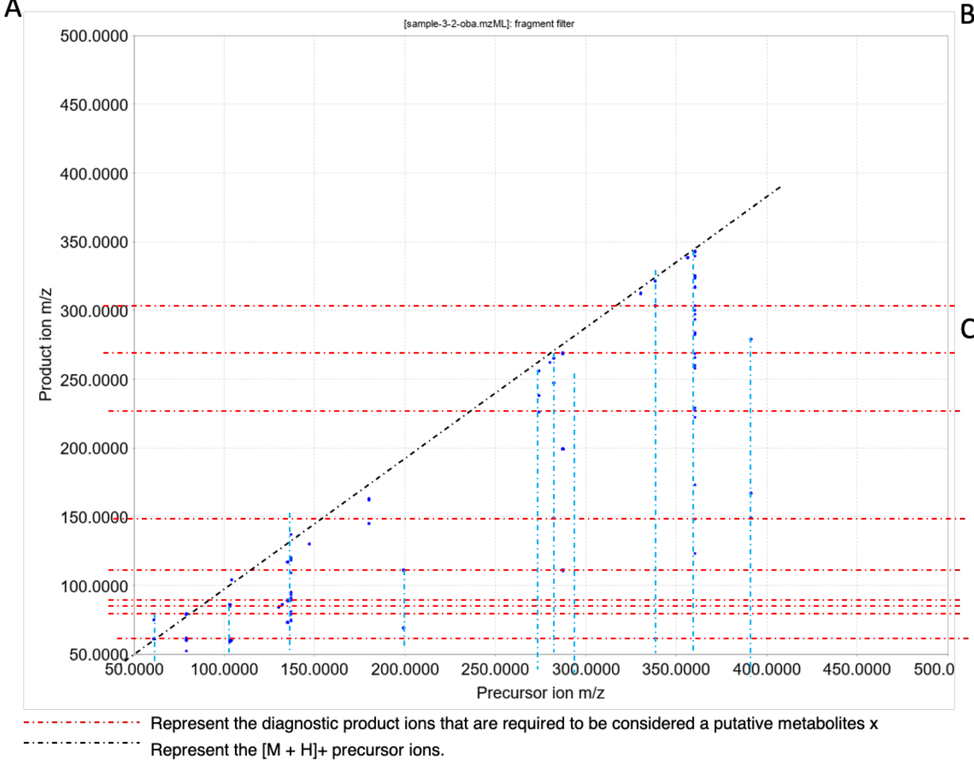

B

	Allium 3			
	RT (min)	MS1 (m/z)	MS2 (m/z)	MS3 (m/z)
1	2.25	104.1058	60.0135	can't detect
2	10.53	330.3329	312.2917	101.9833
3	10.93	282.2759	265.1280	247.2555
4	10.93	356.3483	338.2873	101.9973
5	12.53	391.2800	148.9577	120.9727
6	12.90	338.3378	148.9526	121.0148

C

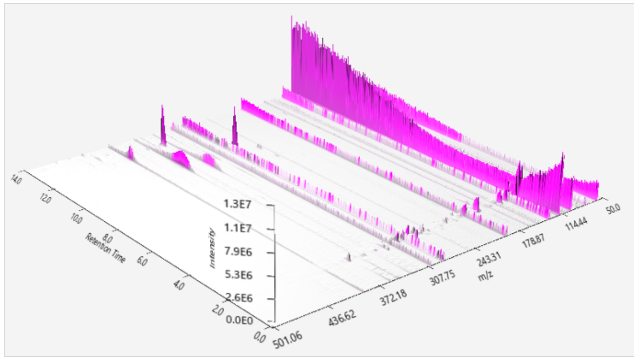

Figure S3. LC-MS profiling of bioactive AL3 extract. (A) Diagnostic Fragmentation Filtering (DFF) plot for metabolites analysis. (B) *m/z* list MS(n=3) of AL3 (B) 3D visualization of MS data of AL3.

A

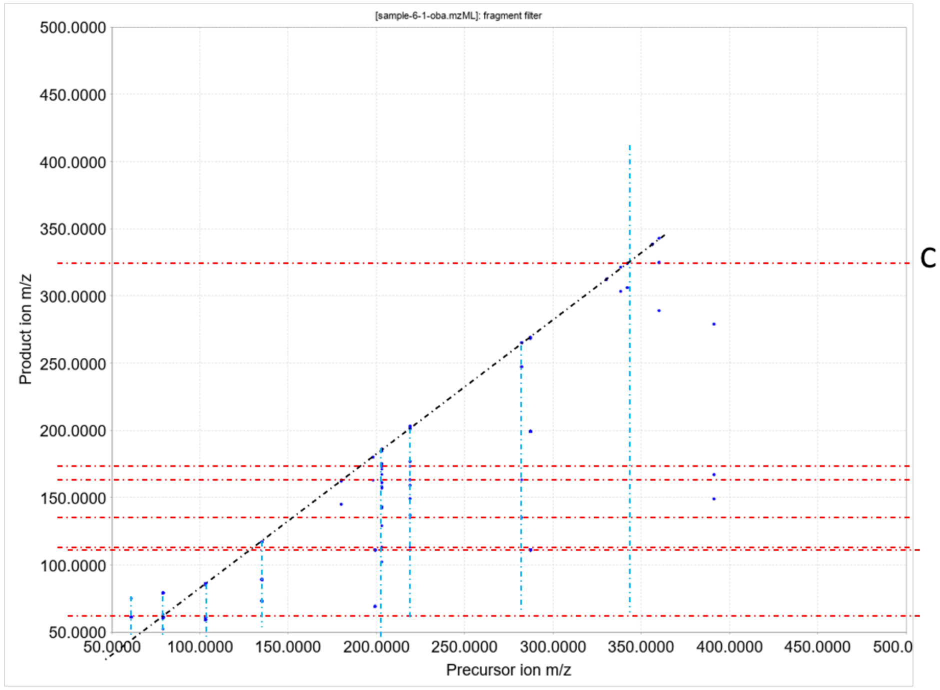

Represent the diagnostic product ions that are required to be considered a putative metabolites x

Represent the [M + H]+ precursor ions.

B

	Allium 6			
	RT (min)	MS1 (m/z)	MS2 (m/z)	MS3 (m/z)
1	1.87	360.1464	325.0564	288.9400
2	1.94	198.0951	179.9797	162.9164
3	1.94	180.0847	162.8774	145.0766
4	2.08	203.0504	185.0148	166.9430
5	2.14	219.0242	158.9557<br>176.7700	98.8628(158.9557)<br>159.1721(176.7700)
6	2.27	104.1058	59.9128	can't detect
7	10.56	330.3330	312.2895	102.0410
8	10.79	356.3484	338.3188	101.9674
9	10.93	282.2759	265.2184<br>247.2157	247.2726(265.2184)<br>163.1118(247.2157)
10	12.48	391.2801	148.9516	121.0154

C

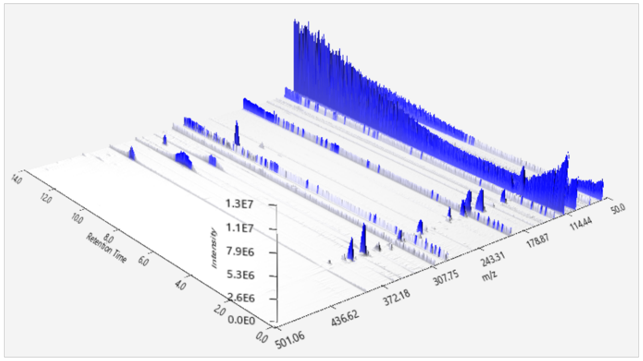

Figure S4. LC-MS profiling of bioactive AL6 extract. (A) Diagnostic Fragmentation Filtering (DFF) plot for metabolites analysis. (B) *m/z* list MS(n=3) of AL6 (B) 3D visualization of MS data of AL6.

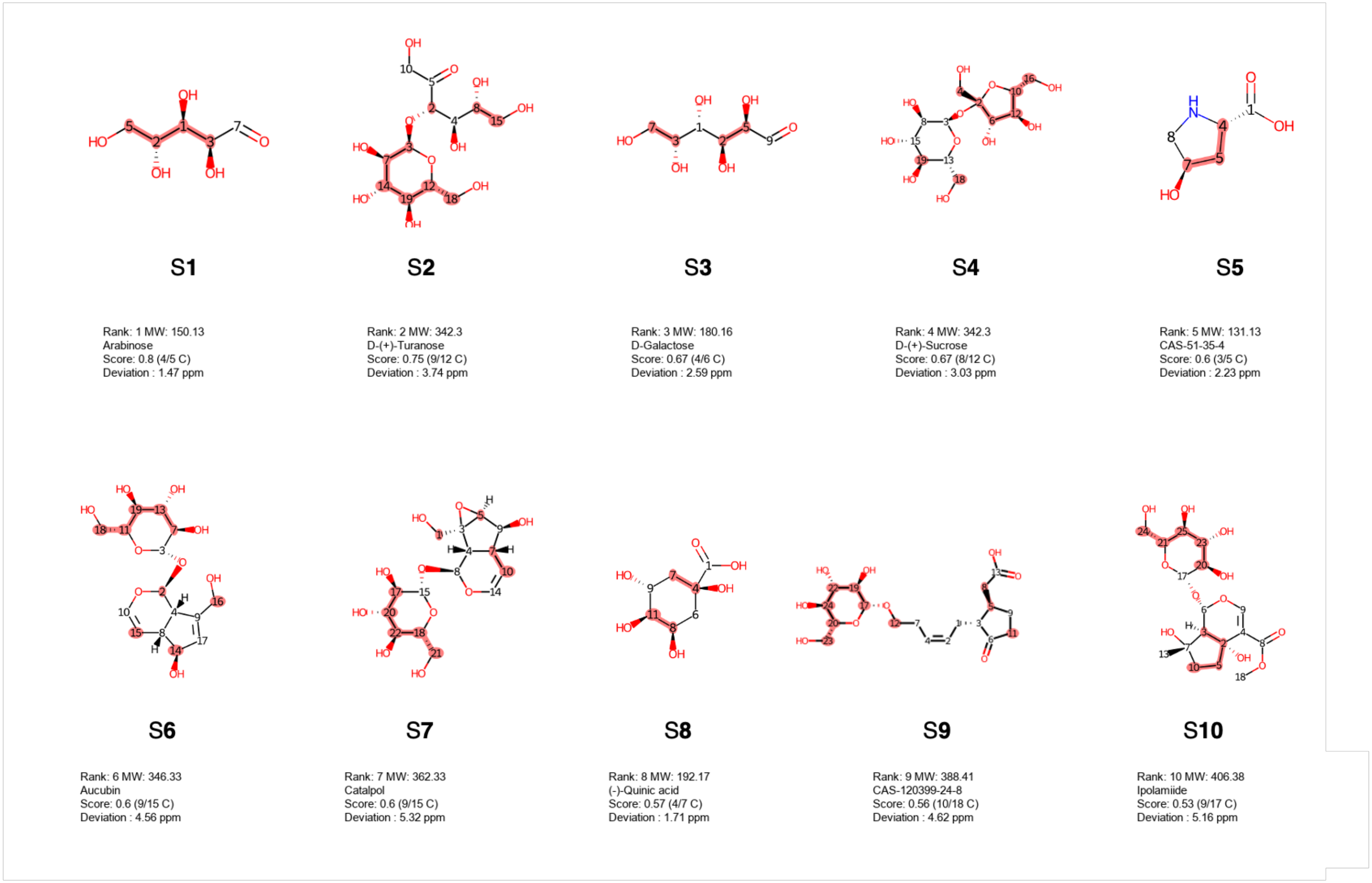

Figure S5. Dereplication analysis from MixONat, structure of top 50 metabolites: compounds S**1**–S**10**.

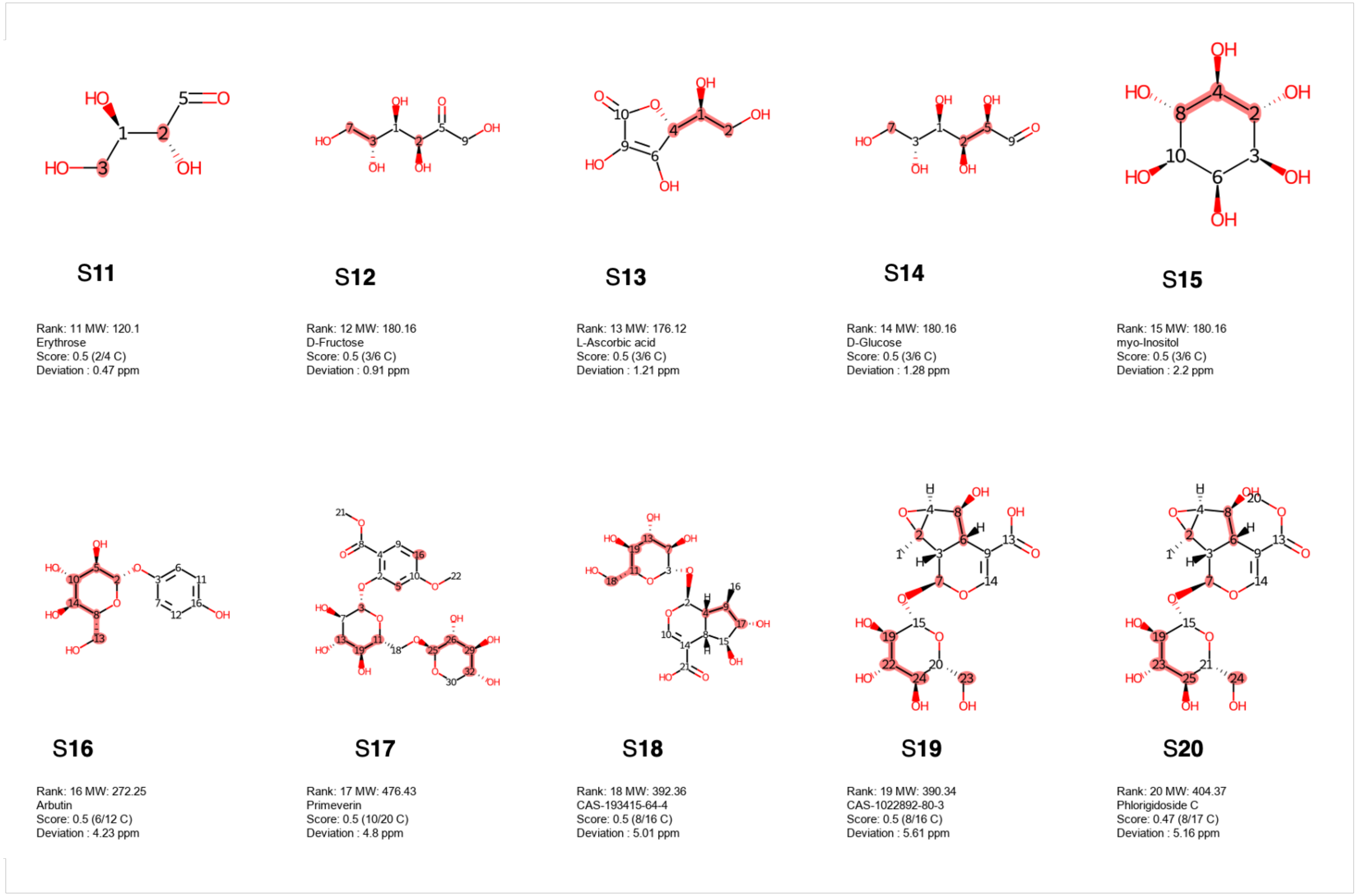

Figure S6. Dereplication analysis from MixONat, structure of top 50 metabolites: compounds S**16**–S**20**.

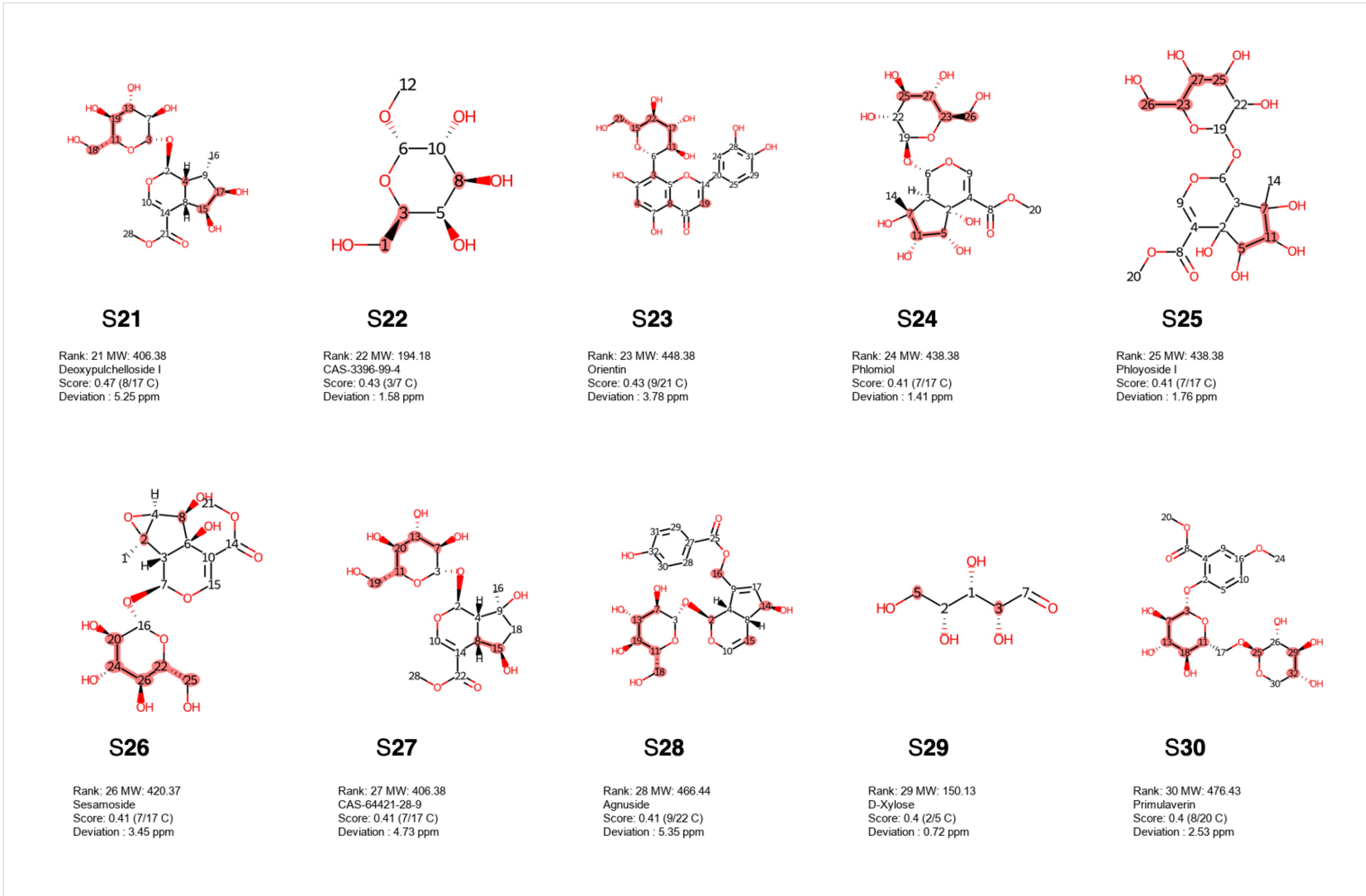

Figure S7. Dereplication analysis from MixONat, structure of top 50 metabolites: compounds S**21**–S**30.**

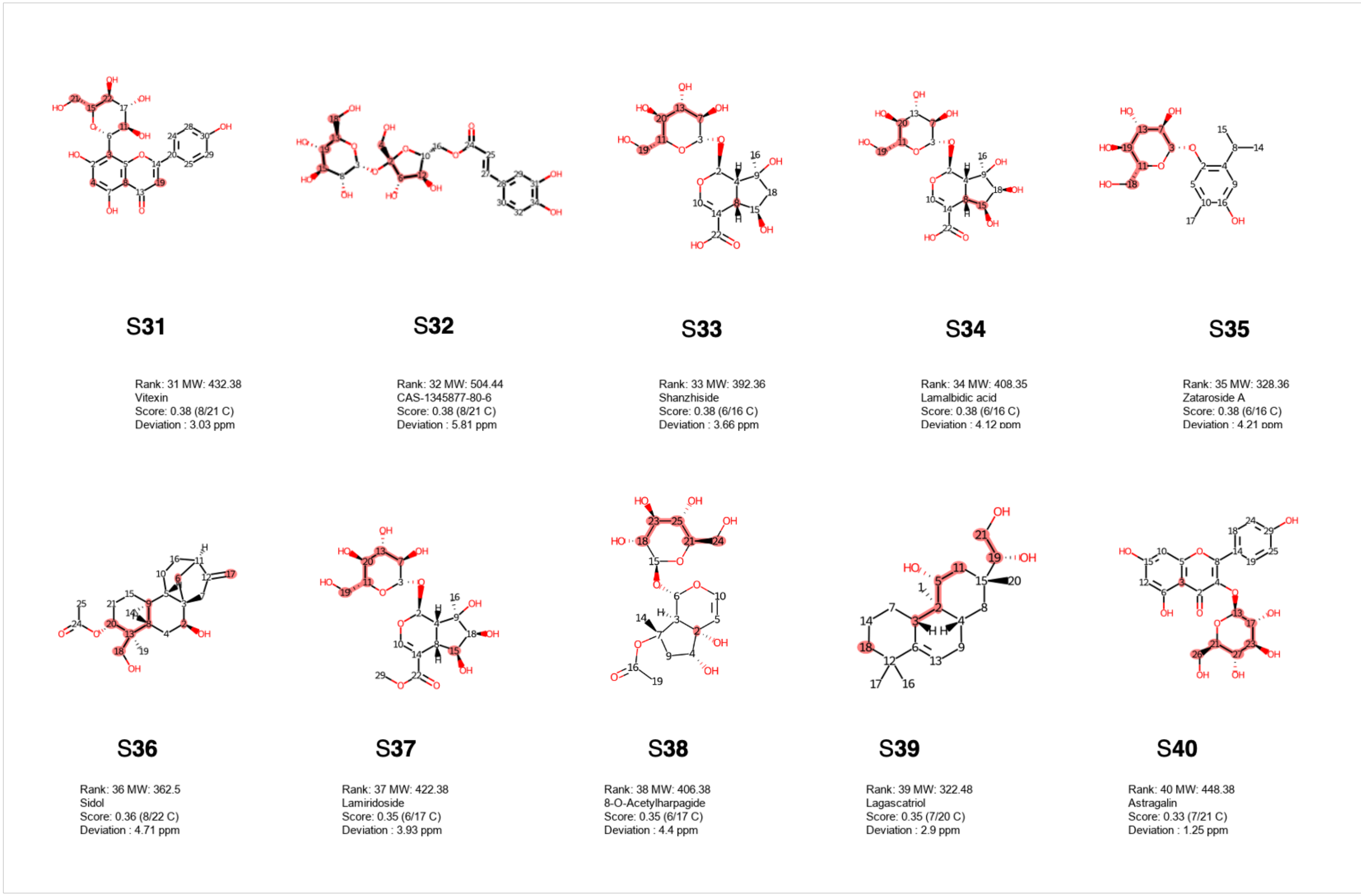

Figure S8. Dereplication analysis from MixONat, structure of top 50 metabolites: compounds S**31**–S**40.**

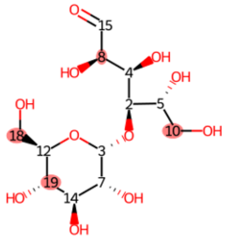

S41

Rank: 41 MW: 342.3
D-(+)-Maltose
Score: 0.33 (4/12 C)
Deviation : 1.29 ppm

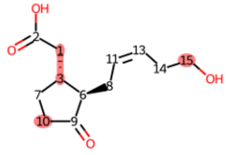

S42

Rank: 42 MW: 226.27
CAS-140631-27-2
Score: 0.33 (4/12 C)
Deviation : 1.42 ppm

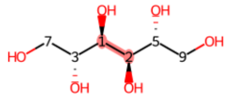

S43

Rank: 43 MW: 182.17
D-Mannitol
Score: 0.33 (2/6 C)
Deviation : 1.62 ppm

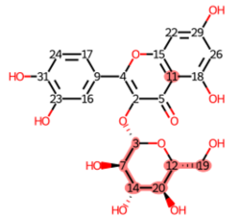

S44

Rank: 44 MW: 464.38
Hirsutrin
Score: 0.33 (7/21 C)
Deviation : 2.02 ppm

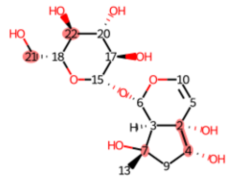

S45

Rank: 45 MW: 364.35
Harpagide
Score: 0.33 (5/15 C)
Deviation : 2.3 ppm

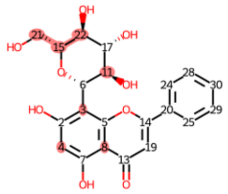

S46

Rank: 46 MW: 416.38
CAS-160880-89-7
Score: 0.33 (7/21 C)
Deviation : 2.97 ppm

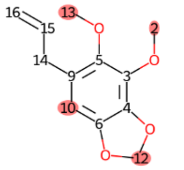

S47

Rank: 47 MW: 222.24
Dillapiole
Score: 0.33 (4/12 C)
Deviation : 3.21 ppm

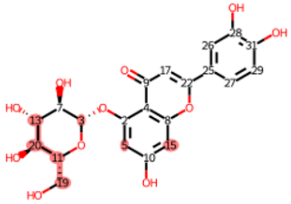

S48

Rank: 48 MW: 448.38
Galuteolin
Score: 0.33 (7/21 C)
Deviation : 3.25 ppm

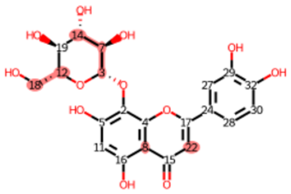

S49

Rank: 49 MW: 464.38
CAS-27686-36-8
Score: 0.33 (7/21 C)
Deviation : 3.47 ppm

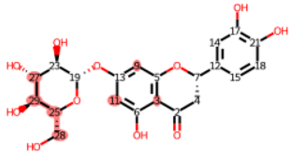

S50

Rank: 50 MW: 450.39
CAS-38965-51-4
Score: 0.33 (7/21 C)
Deviation : 4.51 ppm

Figure S9. Dereplication analysis from MixONat, structure of top 50 metabolites: compounds S**41**–S**50.**

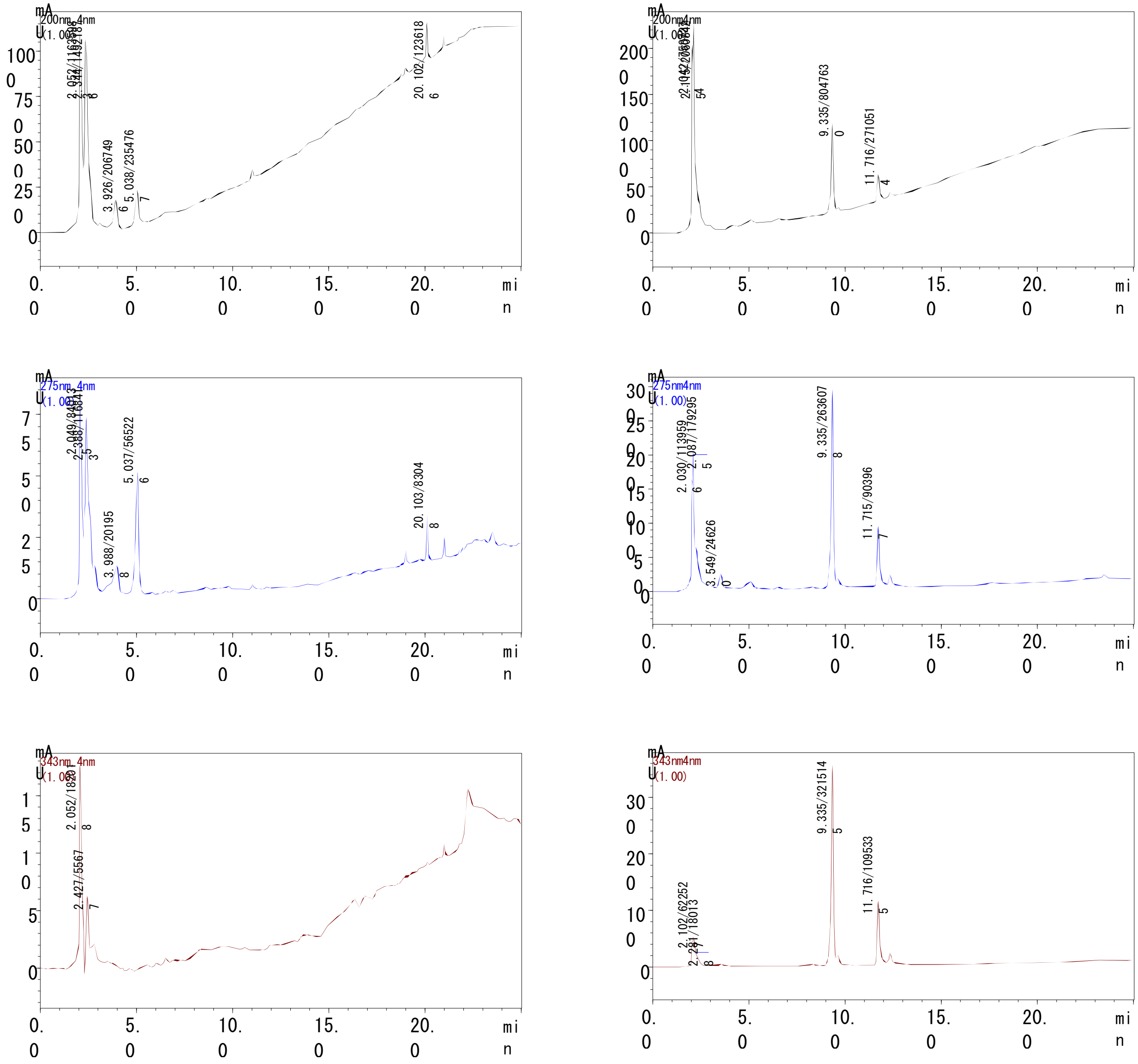

Figure S10. HPLC spectra of AL1 and AL6 (Left: AL1, Right: AL6).

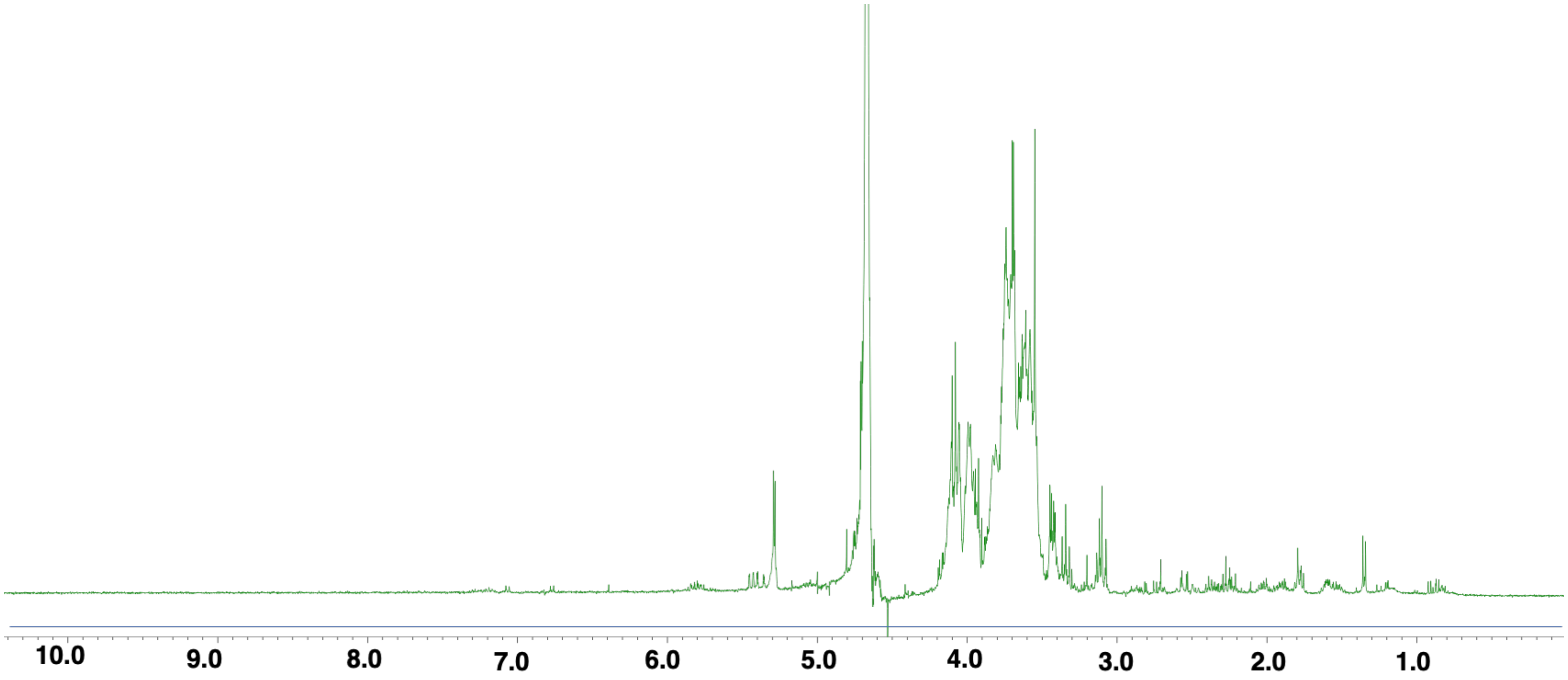

Figure S11. ^{1}H NMR spectrum of AL1 in DMSO.

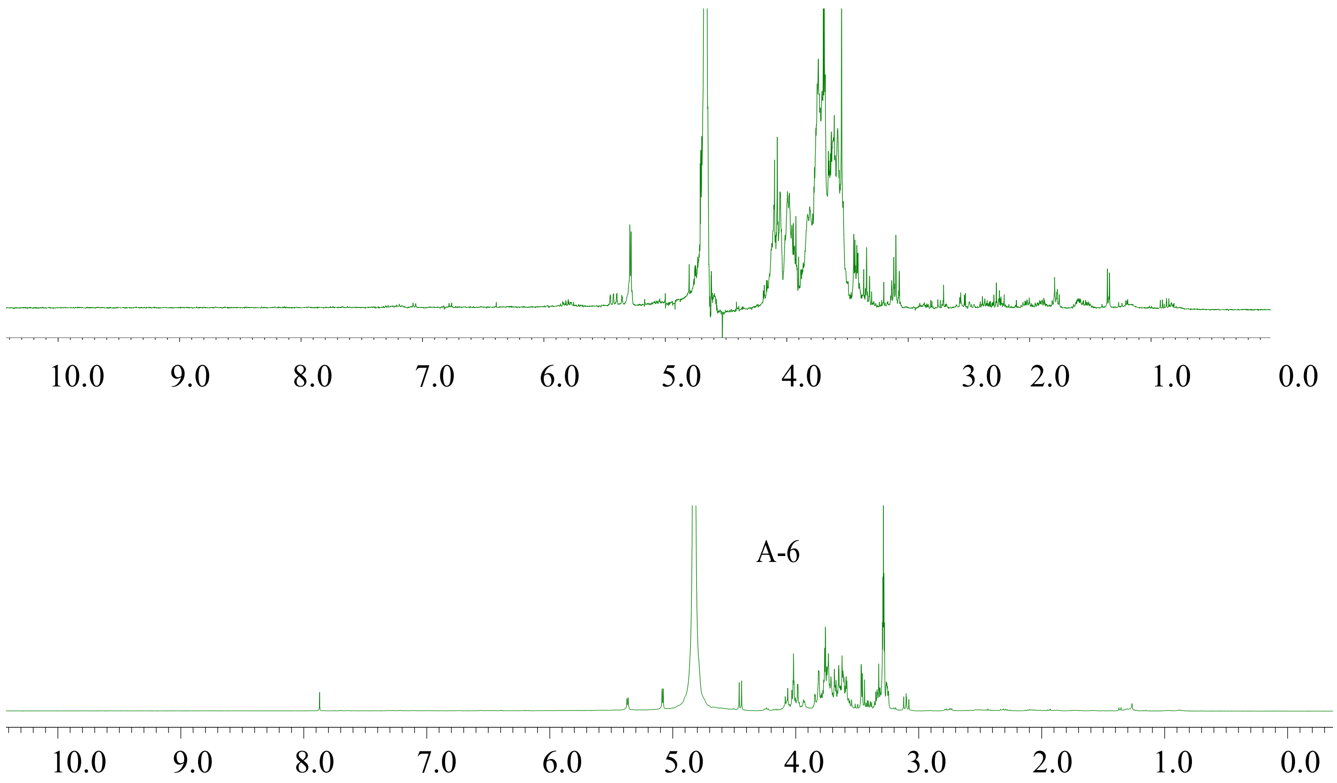

Figure S12. NMR profile of AL1 and AL6.

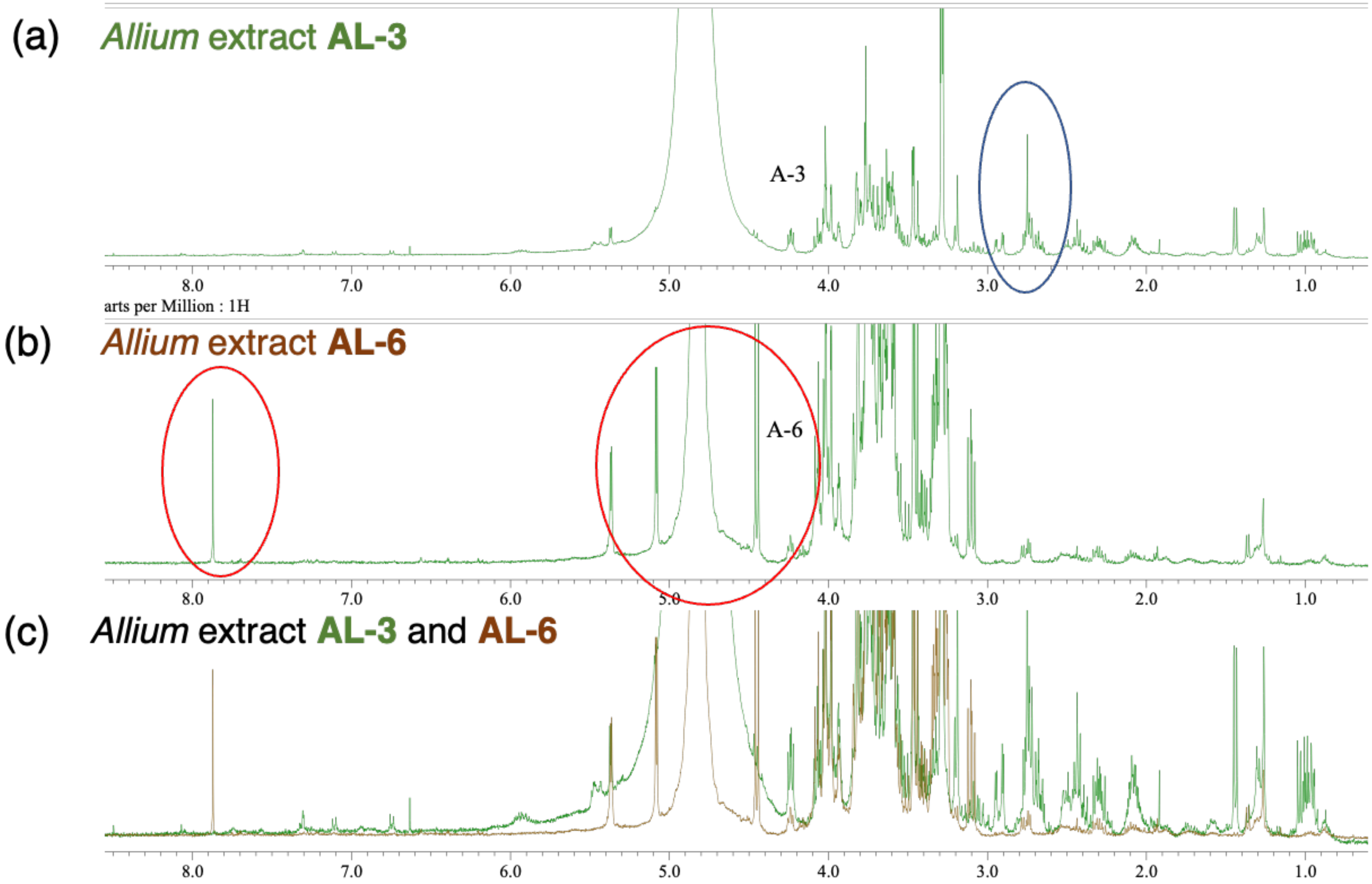

Figure S13. ^{1}H-NMR spectra (a) Spectra group AL3 and (b) Spectra group AL-6 (c) Comparison of ^{1}H-NMR spectra of the AL3 and AL6.

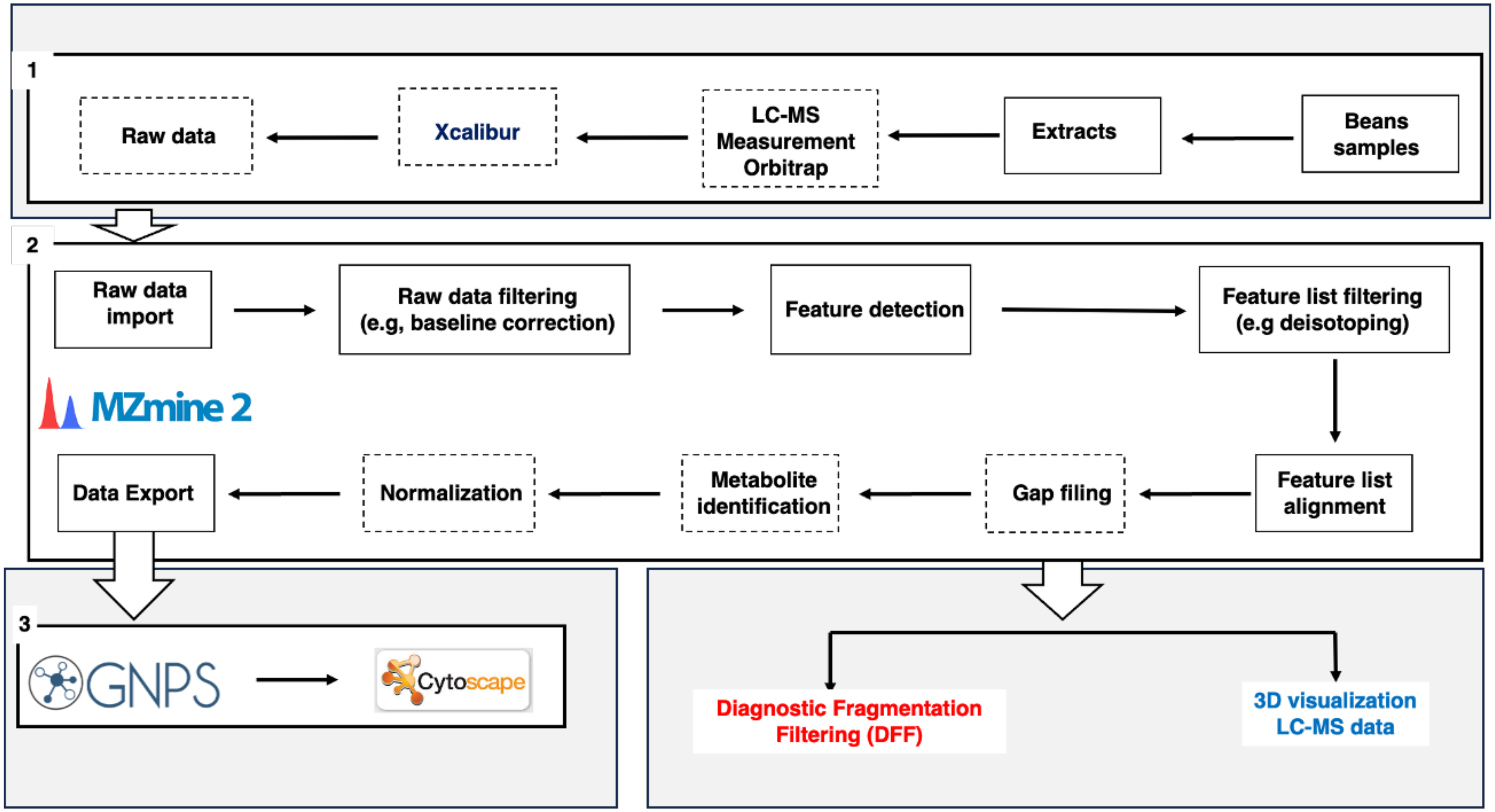

Figure S14. Mixture analysis LC-MS/MS experimental flow

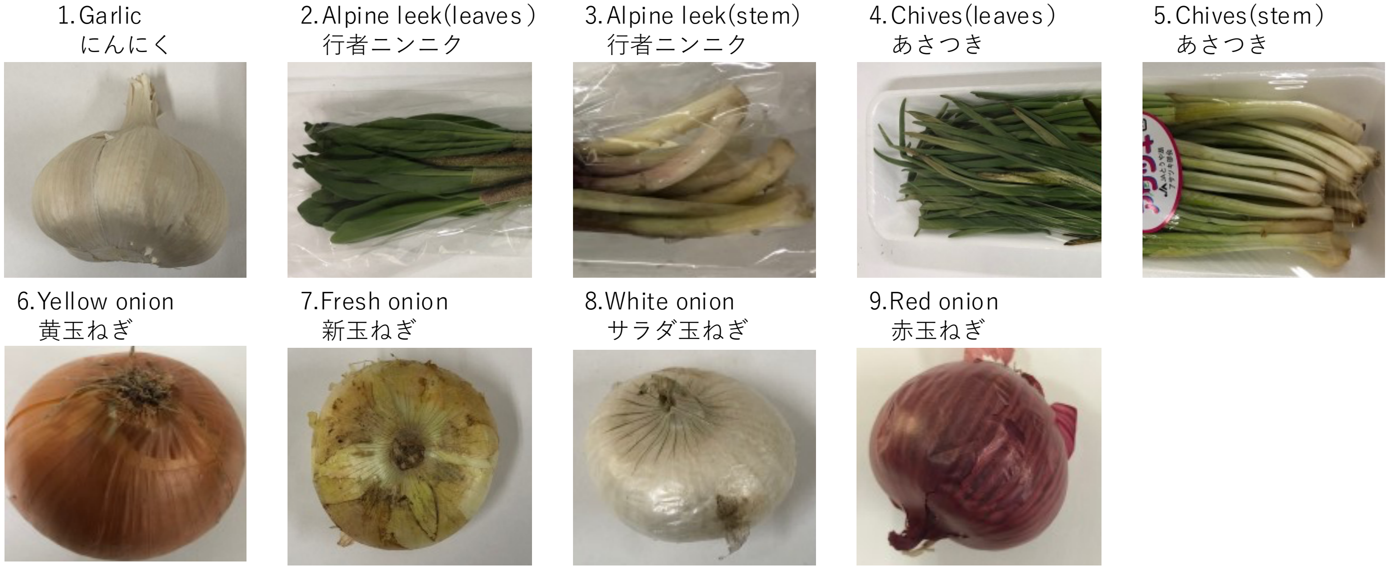

No	Species	Family	Local name	Consumed/used as	The part used in this study
1	*Allium sativum*	*Allium*	Ninniku	cooked, raw	bulb
2	*Allium victorialis*	*Allium*	Gyoujaninnniku	cooked, raw	leaves
3	*Allium victorialis*	*Allium*	Gyoujaninnniku	cooked, raw	stem
4	*Allium schoenoprasum var. foliosum*	*Allium*	Asatuki	cooked, raw	leaves
5	*Allium schoenoprasum var. foliosum*	*Allium*	Asatuki	cooked, raw	stem
6	*Allium cepa*	*Allium*	Kitamanegi	cooked, raw	bulb
7	*Allium cepa*	*Allium*	Sinntamanegi	cooked, raw	bulb
8	*Allium cepa*	*Allium*	Sirotamanegi	cooked, raw	bulb
9	*Allium cepa*	*Allium*	Akatamanegi	cooked, raw	bulb

Picture S1. List and pictures of part of Allium AL1-9 used in the study
